# Novel mechanisms for the removal of strong replication-blocking HMCES- and thiazolidine-DNA adducts in humans

**DOI:** 10.1093/nar/gkad246

**Published:** 2023-04-06

**Authors:** Yohei Sugimoto, Yuji Masuda, Shigenori Iwai, Yumi Miyake, Rie Kanao, Chikahide Masutani

**Affiliations:** Department of Genome Dynamics, Research Institute of Environmental Medicine, Nagoya University, Furo-cho, Chikusa-ku, Nagoya 464-8601, Japan; Department of Molecular Pharmaco-Biology, Nagoya University Graduate School of Medicine, 65 Tsurumai-cho, Showa-ku, Nagoya 466-8550, Japan; Department of Genome Dynamics, Research Institute of Environmental Medicine, Nagoya University, Furo-cho, Chikusa-ku, Nagoya 464-8601, Japan; Department of Molecular Pharmaco-Biology, Nagoya University Graduate School of Medicine, 65 Tsurumai-cho, Showa-ku, Nagoya 466-8550, Japan; Graduate School of Engineering Science, Osaka University, 1-3 Machikaneyama, Toyonaka, Osaka 560-8531, Japan; Forefront Research Center, Graduate School of Science, Osaka University, 1-1 Machikaneyama, Toyonaka, Osaka 560-0043, Japan; Department of Genome Dynamics, Research Institute of Environmental Medicine, Nagoya University, Furo-cho, Chikusa-ku, Nagoya 464-8601, Japan; Department of Molecular Pharmaco-Biology, Nagoya University Graduate School of Medicine, 65 Tsurumai-cho, Showa-ku, Nagoya 466-8550, Japan; Department of Genome Dynamics, Research Institute of Environmental Medicine, Nagoya University, Furo-cho, Chikusa-ku, Nagoya 464-8601, Japan; Department of Molecular Pharmaco-Biology, Nagoya University Graduate School of Medicine, 65 Tsurumai-cho, Showa-ku, Nagoya 466-8550, Japan

## Abstract

Apurinic/apyrimidinic (AP) sites are DNA lesions created under normal growth conditions that result in cytotoxicity, replication-blocks, and mutations. AP sites are susceptible to β-elimination and are liable to be converted to DNA strand breaks. HMCES (5-hydroxymethylcytosine binding, ES cell specific) protein interacts with AP sites in single stranded (ss) DNA exposed at DNA replication forks to generate a stable thiazolidine protein-DNA crosslink and protect cells against AP site toxicity. The crosslinked HMCES is resolved by proteasome-mediated degradation; however, it is unclear how HMCES-crosslinked ssDNA and the resulting proteasome-degraded HMCES adducts are processed and repaired. Here, we describe methods for the preparation of thiazolidine adduct-containing oligonucleotides and determination of their structure. We demonstrate that the HMCES-crosslink is a strong replication blocking adduct and that protease-digested HMCES adducts block DNA replication to a similar extent as AP sites. Moreover, we show that the human AP endonuclease APE1 incises DNA 5′ to the protease-digested HMCES adduct. Interestingly, while HMCES-ssDNA crosslinks are stable, the crosslink is reversed upon the formation of dsDNA, possibly due to a catalytic reverse reaction. Our results shed new light on damage tolerance and repair pathways for HMCES-DNA crosslinks in human cells.

## INTRODUCTION

DNA apurinic/apyrimidinic (AP) sites are a major DNA lesion generated under normal growth conditions as a result of spontaneous or DNA glycosylase catalysed hydrolysis of *N*-glycosidic bonds between the base and deoxyribose backbone (1–4). *N*-glycosidic bonds with purines, especially guanine, are liable to undergo spontaneous hydrolysis, while base modifications, such as alkylation, can destabilise the *N*-glycosidic bond and enhance spontaneous hydrolysis (2,3). Consequently, approximately 10 000 AP sites are generated per cell per day (5,6).

AP sites are cytotoxic replication-blocking lesions with the potential to be mutagenic due to their non-coding nature (1,2,7,8). AP sites are largely repaired by base excision repair (BER); however, a fraction of AP sites persist, as demonstrated by the steady state of 50 000–200 000 AP sites in mammalian cells (9). DNA damage tolerance (DDT) pathways, such as translesion DNA synthesis (TLS) and template switching (TS), play important roles in protecting cells against this cytotoxicity (10–12). Mutagenesis provoked by AP sites in eukaryotes has been studied using a wide variety of experimental systems. The transformation of eukaryotic cells with AP site-containing plasmids or oligonucleotides has demonstrated that dCMP is preferentially inserted opposite an AP site (termed C-rule insertion) in a manner dependent on REV1 dCMP transferase activity (13–17). Importantly, the mutation spectrum of tetrahydrofuran (THF), a stable synthetic AP site analogue, is different from that of natural AP sites, in which insertion of dAMP opposite THF is favoured over insertion of dCMP (15–17). The contribution of dCMP insertion opposite endogenous AP sites to TLS was for a long time uncertain because this incorporation opposite AP sites generated by guanine depurination is undetectable due to restoration of the wild type sequence. However, an elegant study by the Demple group has shown that dCMP is, indeed, frequently incorporated opposite AP sites produced by mutant glycosylases in yeast cells (10,18). This is consistent with the work conducted by the Jinks-Robertson group using a well-designed yeast genetic system to examine nucleotides incorporated opposite AP sites (19).

AP sites are present as an equilibrium of furanose and aldehyde forms in solution. The aldehyde form is susceptible to base-catalysed β-elimination and thus, AP sites can be converted to DNA strand breaks (7). Recently, HMCES (5-hydroxymethylcytosine binding, ES cell specific), a replication fork-associated protein (20,21), was discovered to interact with the aldehyde form of AP sites and generate stable thiazolidine protein-DNA crosslinks (20,22–24). The crosslinking reaction is specific to AP sites in single-stranded DNA (ssDNA) (20,22,23). Crosslinked AP sites exposed to replication forks prevent spontaneous and nuclease-dependent breakage of the fork, protecting cells from the toxic effects of AP sites (20,25,26). The HMCES-crosslink prevents DNA synthesis by translesion DNA polymerases, REV1-pol ζ, pol κ, and probably pol η, resulting in the suppression of AP site-induced mutagenesis (20,21,26). However, the process involved is poorly characterised biochemically.

The crosslink activity of HMCES is located in its SRAP (SOS response associated peptidase) domain (20,22–24,27). The peptidase activity of SRAP removes the N-terminal methionine to expose cysteine at position 2, which then forms a thiazolidine link with the ring-opened AP site (20,22,23).

HMCES was previously referred to as C3orf37 after quantitative mass-spectrometry-based proteomics revealed that it was a 5-hydroxymethylcytosine-binding protein in mouse embryonic stem cell extracts (28). Later, another study reported that HMCES binds to oxidised 5-methylcytosine-containing DNA, incises DNA 3′ to 5-hydroxymethylcytosine, and contributes to the erasure of DNA methylation during embryogenesis (29). However, its purported function as an epigenetic regulator was not confirmed by other studies (20,30) and thus it was considered that HMCES might be a misnomer (8). Interestingly, replication-independent functions of HMCES were also reported; HMCES prevents deletions during somatic hypermutation and facilitates alternative-end-joining pathways during class switch recombination in B cells (30,31). The former required the crosslinking activity of HMCES, but the later did not, although its DNA binding activity was still needed; however, the underlying mechanisms were unclear (30,31). In addition to the repair functions of HMCES mentioned above, a recent report suggested that HMCES might be involved in early embryonic development in mouse and *Xenopus laevis* via a transcriptional regulatory function (32).

Studies in *Xenopus laevis* egg extracts demonstrated the degradation of HMCES crosslinked to ssDNA by replication-coupled protease SPRTN and subsequent TLS across the degraded HMCES-crosslinks (33,34). In human cells, one report suggested that the HMCES-ssDNA crosslink is resolved by proteasome-mediated degradation (20); however, it was unclear how these crosslinks are processed and repaired to restore intact dsDNA in humans. In the present study, we examined the functions of a human replicative DNA polymerase, pol δ, Y-family translesion DNA polymerases, an X-family DNA polymerase, pol β, AP endonucleases, and a phosphodiesterase, TDP1, in the processing and repair of thiazolidine-DNA lesions.

## MATERIALS AND METHODS

### Plasmids

*HMCES* and *APE1* cDNAs were amplified from a HeLa cDNA library and cloned into the *Nde* I-*Xho* I sites of pET-20b(+), generating pET20-HMCES-his and pET20-APE1, which express C-terminally histidine tagged HMCES (HMCES-his) and intact APE1, respectively. *APE2* cDNA was amplified from a HeLa cDNA library and a fragment encoding the catalytic domain (amino acids 1–361) was cloned into the *Nde* I-*Xho* I sites of pET15b (Novagen), generating pET15-his-APE2(cat). The entire coding region was sub-cloned into pBAD22 (35), generating pBAD-his-APE2(cat), which expresses a fusion protein composed of a histidine tag covalently linked to the N-terminus of APE2(cat) (his-APE2(cat)). *TDP1* cDNA was amplified from a HeLa cDNA library and cloned into pBAD22 in the same way as for *APE2* cDNA, generating pBAD-his-TDP1, which expresses a fusion protein composed of a histidine tag covalently linked to the N-terminus of TDP1 (his-TDP1). For expression in human cells, FLAG-tagged *APE2* and a D277A catalytic-dead mutant (36) were cloned into the *Nhe* I-*Bam*H I sites of pIRESneo2 (Clontech), generating pIRESneo2-FLAG-APE2 and pIRESneo2-FLAG-APE2(D277A), respectively. A DNA fragment containing the *Escherichia coli* uracil-DNA glycosylase gene (*ung*) was amplified from DH5α genomic DNA and cloned into the *Nde* I-*Xho* I sites of pET-20b(+), generating pET-UNG.

### Proteins

*Escherichia coli* exonuclease III (ExoIII) was purchased from Takara Bio inc (2170A). *E. coli* uracil-DNA glycosylase (UDG), endonuclease III (EndoIII), and endonuclease IV (EndoIV) were purchased from New England Biolabs (M0280L, M0268S and M0304S, respectively).

HMCES-his was purified as follows. *E. coli* BL21 (DE3) harbouring pET20-HMCES-his and pMStRNA1 (37) was grown at 15°C in LB medium containing ampicillin (250 μg/ml) and kanamycin (30 μg/ml) to *A*_600_ 0.6, after which recombinant HMCES-his expression was induced by adding 0.2 mM isopropyl β-d-1-thiogalactopyranoside (IPTG). After centrifugation, the cells were lysed as described previously (38) and recombinant HMCES-his was purified from the cell lysates by chromatography at 4°C on Ni^2+^-charged HiTrap Chelating HP, HiTrap Heparin HP, HiTrap SP HP, and Superdex 200 columns (GE Healthcare). Mutant HMCES-his (W290G/L291A), HMCES^WG/LA^ (29), was purified in parallel.

APE1 was purified as described previously (39) with several modifications. Briefly, *E. coli* BL21 (DE3) harbouring pET20-APE1 was grown in LB medium containing ampicillin (250 μg/ml) at 15°C and recombinant APE1 was expressed by adding IPTG at 0.2 mM. Cell lysates were fractionated by ammonium sulphate precipitation and then APE1 was purified by chromatography at 4°C using phosphocellulose P11 (Whatman), RESOURCE S, HiTrap Q HP, HiTrap Heparin HP, and Superdex 200 columns (GE Healthcare).

his-APE2(cat) was purified as follows. The *E. coli* ExoIII/EndoIV-deficient strain RPC501 (40) (a kind gift from Dr T. Nunoshiba) harbouring pBAD-his-APE2(cat) was grown in LB medium containing ampicillin (250 μg/ml) and kanamycin (30 μg/ml) at 15°C, and recombinant his-APE2(cat) was expressed by adding l-arabinose at 0.001%. his-APE2(cat) was purified from cell lysates by chromatography at 4°C on Ni^2+^-charged HiTrap Chelating HP, HiTrap Heparin HP, HiTrap Q HP, and Superdex 200 columns (GE Healthcare).

his-TDP1 was purified as follows. RPC501 (40) harbouring pBAD-his-TDP1 was grown in LB medium containing ampicillin (250 μg/ml) and kanamycin (30 μg/ml) at 15°C, and recombinant his-TDP1 was expressed by adding _L_-arabinose at 0.001%. his-TDP1 was purified from cell lysates by chromatography at 4°C on Ni^2+^-charged HiTrap Chelating HP, HiTrap Heparin HP, and Superdex 200 columns (GE Healthcare).

UDG was purified for the large-scale preparation of AP site-containing oligonucleotides. *E. coli* BL21 (DE3) harbouring pET20-UNG was grown in LB medium containing ampicillin (250 μg/ml) at 15°C. Recombinant UDG was produced by the addition of IPTG at 0.2 mM and purified from cell lysates by chromatography at 4°C using HiTrap Q FF, HiTrap Heparin HP, HiTrap Benzamidine FF (high sub), HiTrap Capto adhere, HiTrap Phenyl HP, and Superdex 200 columns (GE Healthcare).

Recombinant human pol β, pol δ, pol η, pol ι, his-pol κ (hereafter described as pol κ), REV1 and FLAG-RAD18-his-RAD18-RAD6 complex were purified as described previously (38,41–44). Exonuclease defective pol δ (pol δ^exo−^) was generated by introducing D316A and E318A double mutations (45). Protein concentrations were determined using the Bio-Rad protein assay (Bio-Rad) using BSA (Bio-Rad) as a standard.

FLAG-tagged full-length APE2, FLAG-APE2 and a D277A mutant were partially purified as follows. The human embryonic kidney cell line HEK293 was cultured in Dulbecco's modified Eagle's medium (DMEM) (FUJIFILM Wako Pure Chemical Corporation) supplemented with 10% fetal bovine serum (Sigma-Aldrich) and 1 × Penicillin–Streptomycin Mixed Solution (Nacalai Tesque). The cells were then transfected with pIRESneo2-FLAG-APE2 or pIRESneo2-FLAG-APE2(D277A) using the ViaFect transfection reagent (Promega), incubated for 24 hours, and lysed in Lysis buffer (20 mM Tris–HCl (pH 7.6), 150 mM NaCl, 10% glycerol, 0.5% NP-40, 5 mM β-mercaptoethanol, cOmplete Protease Inhibitor Cocktail (Merck), and PhosSTOP (Merck)). Crude lysates were centrifuged at 4°C and supernatants were incubated with anti-FLAG affinity gel (Sigma-Aldrich) for 3 hours at 4°C, following which the beads were centrifuged, washed three times with Lysis buffer, and eluted with 0.2 mg/ml FLAG peptide (Sigma-Aldrich) in Lysis buffer. The concentration of purified protein was determined by western blot with an anti-FLAG M2 monoclonal antibody (Sigma-Aldrich, F3165) using a purified FLAG-RAD18-his-RAD18-RAD6 ternary complex of known concentration (44) as a standard.

### Oligonucleotides

**Figure 1.**
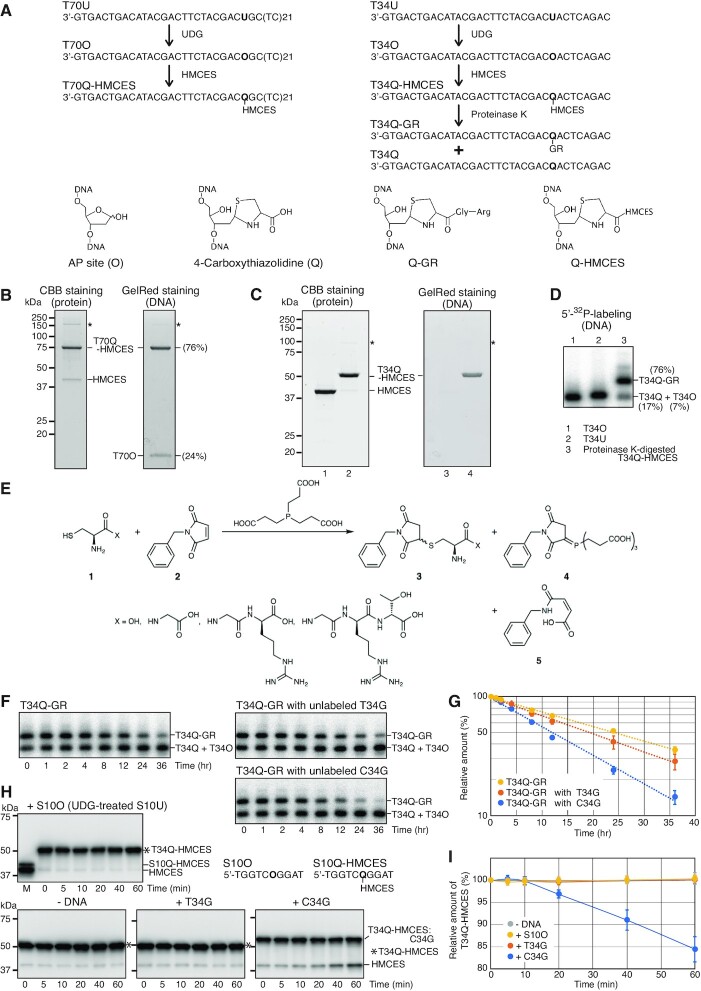
HMCES-crosslink- and thiazolidine adduct-containing DNA. (**A**) Strategy for the preparation of HMCES-crosslink- and thiazolidine adduct-containing DNA. Chemical structures of the lesions are shown. O and Q indicate AP sites and the 4-carboxythiazolidine adduct, respectively. (**B, C**) SDS-PAGE analysis of purified HMCES-crosslinked oligonucleotides. The purified T70Q-HMCES (B), HMCES (C, lanes 1 and 3) and T34Q-HMCES (C, lanes 2 and 4) were analysed by 15% SDS-PAGE. The gels were fixed with methanol and stained with a fluorescence dye, GelRed. After the detection of fluorescence, the same gels were stained with CBB. The AP site-containing oligonucleotide T70O is stable during SDS-PAGE (data not shown). T34O was undetectable in this experiment (data not shown). Asterisks indicate conformationally different forms of HMCES-crosslinked DNA. (**D**) Urea–PAGE analysis of the proteinase K-digested T34Q-HMCES. The digested T34Q-HMCES was 5′-labelled with ^32^P and analysed by 10% urea–PAGE (lane 3). Relative amounts of each product are shown in parentheses. T34O (lane 1) and T34U (lane 2) were loaded as controls. (**E**) Derivatisation of cysteine and peptides. (**F, G**) Stability of the Q-GR adduct. 5′-^32^P-labelled T34Q-GR containing DNA was incubated in TE (10 mM Tris–HCl pH 7.5, 1 mM EDTA) at 37°C in the presence or absence of the indicated unlabelled DNA. Aliquots were withdrawn at the indicated times and resolved on 10% urea–PAGE (F). The relative radioactivity of each of T34Q-GR fractions was measured and averages of three independent experiments are plotted with SD in the graph (G). T34G is on the same strand as T34Q while C34G is on the complementary strand to T34Q (Supplementary Table S1). (**H, I**) Stability of the HMCES-crosslink. The purified T34Q-HMCES was incubated at 30°C in the presence or absence of the indicated DNA. Aliquots were withdrawn at the indicated times and analysed by western blot with an anti-HMCES antibody (H). T34Q-HMCES:C34G indicates T34Q-HMCES annealed to C34G. A crosslink reaction between HMCES and S10O was loaded in lane M as a control. The relative amounts of T34Q-HMCES fractions were measured and averages of three independent experiments are plotted with SD in the graph (I).

Oligonucleotides used in this study (Supplementary Table S1) were purchased from Fasmac Co., Ltd. To generate AP sites (labelled O in the sequence), oligonucleotides containing uracil, T70U, T34U, and S10U, were treated with 0.5 units of UDG (NEB) per pmol of DNA at 30°C for 20 min unless otherwise indicated. The resultant oligonucleotides were respectively designated as T70O, T34O and S10O, where O indicates the AP site (Figure 1A and H).

### Generation of HMCES-linked DNA constructs

A 70-mer (T70U) or 34-mer (T34U) oligonucleotide (8 nmol) was incubated with partially purified HMCES-his (30 nmol) eluted from a HiTrap SP HP column in 50 mM HEPES–NaOH (pH 7.5), 200 mM NaCl, 10% glycerol, 10 mM β-mercaptoethanol, and UDG (NEB 4000 unts) at 30°C for 20 min. The crosslinked HMCES-his oligonucleotides (referred to as T70Q-HMCES and T34Q-HMCES, respectively, where Q indicates the 4-carboxythiazolidine adduct; Figure 1A), were purified by chromatography at 4°C on Ni^2+^-charged HiTrap Chelating HP and Mono Q columns.

To obtain proteinase K-digested T34Q-HMCES, T34U (111 nmol) was incubated with 78.4 nmol of purified UDG at 30°C for 20 min. Subsequently, 222 nmol of HMCES^WG/LA^ was added in the presence of 50 mM HEPES–NaOH (pH 7.5), 300 mM NaCl, 10% glycerol, 10 mM β-mercaptoethanol, and 0.0125% TritonX100 at 37°C for 1 h. T34Q-HMCES^WG/LA^ was purified as described above and treated with proteinase K at 37°C for 9 h. The resultant 4-carboxythiazolidine adduct-containing DNA was purified by phenol-chloroform extraction, chloroform extraction, and ethanol precipitation.

### Preparation of *N*-benzylmaleimide-derivatised peptides as reference materials

Cysteine and the dipeptide (CG) were purchased from Sigma-Aldrich, and the tripeptide (CGR) and the tetrapeptide (CGRT) were obtained from GenScript. Hereafter, the respective *N*-benzylmaleimide (NBM)-derivatised peptides are referred to as Bn-Cys, Bn-CG, Bn-CGR, and Bn-CGRT. Cysteine, CG, CGR and CGRT (100 nmol) were mixed with tris(carboxyethyl)phosphine hydrochloride (TCEP·HCl, 100 nmol) in water (35 μl), and after 1 h, a 50 mM solution of NBM in ethanol (5 μl) was added. The mixtures were kept at 25°C overnight, and then aliquots were analysed by HPLC (Supplementary Figure S4A, S4C, S4E, and S4G). Eluants A and B were 0.1% formic acid in water and 0.1% formic acid in acetonitrile containing 5% water, respectively. A GL Sciences Inertsil ODS-3 5 μm column (4.6 × 250 mm) was used on a Gilson gradient-type analytical system at a flow rate of 1.0 ml/min with a linear gradient of 10–40% B for 20 min, and the data were acquired using a Waters 2998 photodiode array detector and Empower 3 software. The products were purified under the same conditions by monitoring absorption at 260 nm using a Gilson 151 UV/VIS detector. The eluates were dried on a Thermo Scientific Savant SpeedVac concentrator, and after formic acid was removed by co-evaporation with water, the derivatised cysteine and peptides were dissolved in 20% aqueous acetonitrile. The absorbance at 260 nm of each solution was measured, and the concentrations of the derivatives were calculated using the molar extinction coefficient of benzyl alcohol (ϵ_260_ = 275 l/(mol·cm)) (46).

### Derivatisation of the peptides cleaved from the proteinase K-digested T34Q-HMCES

Since proteinase K-digested T34Q-HMCES was considered to be a mixture of T34Q and T34Q conjugating with N-terminal peptide(s) of HMCES, hereafter proteinase K-digested T34Q-HMCES is referred to as T34Q-X. The T34Q-X sample (15 nmol) was desalted using a NAP-10 column (Cytiva), and the eluate was dried on a SpeedVac concentrator. The residue was dissolved in 10 mM HEPES–NaOH (pH 7.5, 100 μl), and after heating at 95°C for 10 min, the solution was loaded onto a NAP-5 column (Cytiva) equilibrated with water. The column was washed with 1.4 mL of water, and the low-molecular-weight compounds were eluted with 3 mL of water. The eluate was divided into two and dried in a SpeedVac concentrator. One of the residues was dissolved in 160 mM sodium phosphate (pH 6.0, 25 μl) and mixed with 10 mM TCEP·HCl (10 μl). After 1 h, 50 mM NBM in ethanol (5 μl) was added, and the mixture was kept at 25°C overnight. The derivatised peptides were purified by HPLC, as described for the reference materials. Eluates were collected at 5–8, 12–13.5 and 14.5–16.5 min, which contained Bn-CGRT plus Bn-CGR, Bn-CG, and Bn-Cys, respectively. The combined eluates were dried in a SpeedVac concentrator, and formic acid was removed by co-evaporation with water. A blank sample was prepared using the constituents of the above-mentioned derivatisation reaction and the following procedure without the materials cleaved from the T34Q-X sample.

### Preparation of standard solutions of NBM-derivatised peptides and sample solution of T34Q-X for LC–MS analysis

LC–MS grade acetonitrile, ultrapure water, and formic acid were obtained from FUJIFILM Wako Pure Chemical Corporation. The NBM-derivatised peptide reference materials were used to identify and determine those in the T34Q-X sample by LC–MS analysis. Each reference material, Bn-Cys, Bn-CG, Bn-CGR, or Bn-CGRT, was dissolved in acetonitrile/water (50:50) (100 μM), and then standard mixed solutions were prepared using acetonitrile/water (10:90) (0–1 μM) or acetonitrile/water (25:75) (0–0.1 μM). These working standard solutions were used to construct calibration curves and perform spiked tests. The derivatised sample obtained from the T34Q-X sample was dissolved in 100 μl of acetonitrile/water (50:50) and then diluted 10-fold using acetonitrile/water (5:95) for the determination of Bn-Cys and Bn-CGR, or diluted 2-fold using water for the determination of Bn-CG and Bn-CGRT. The acetonitrile/water solvent used to dissolve reference compounds or sample contained formic acid at a final concentration of 0.1%. Each diluted standard solution or the T34Q-X sample solution was mixed by vortexing and then placed in vials with a deactivated vial insert (Agilent Technologies). Two microliters of the standard solutions or the T34Q-X sample solution were injected into the LC–MS system for analysis.

### LC–MS analysis of NBM-derivatised peptides

LC–MS analysis was performed on a quadruple time-of-flight (QTOF) mass spectrometer (X500R, Sciex) coupled to a UPLC system (ACQUITY UPLC H-class plus, Waters). An InertSustain AQ-C18 column (2.1 i.d. × 100 mm, 1.9 μm, GL Sciences) was used for chromatographic separation at ambient temperature. The eluent system was composed of acetonitrile/water (5:95) containing 0.1% (v/v) formic acid (A) and acetonitrile containing 0.1% (v/v) formic acid (B). The flow rate was 0.2 ml/min. The gradient system was as follows: 0% of eluent B for 0–1 min, 20% of eluent B for 20 min, 95% of eluent B for 21–22 min, and 0% of eluent B for 23–30 min for equilibration. The mass spectrometer was operated in positive electrospray ionisation mode with a capillary voltage of 5500 V. The source temperature was 350°C and the declustering potential was 80 V. The data acquisition in TOF mode with a mass range of *m/z* 100–700 was used for the determination of the NBM-derivatised peptides. Mass calibration with an external standard was performed every 2 hours to maintain mass accuracy during analysis. The determination was carried out using the peak area detected in each extracted ion chromatogram, where the protonated molecules [M + H]^+^, *m/z* 309.091 for Bn-Cys and *m/z* 366.112 for Bn-CG, and doubly-charged protonated molecules [M + 2H] ^2+^, *m/z* 261.611 for Bn-CGR and *m/z* 312.134 for Bn-CGRT, were chosen for the extracted ions resulting in the highest intensity. Tandem mass spectrometry (MS/MS) measurement by collision-induced dissociation was performed to confirm the identification of NBM-derivatised peptides in the T34Q-X sample. The precursor ions with a 1 Da mass window for MS/MS measurements were set to [M + H] ^+^ or [M + 2H] ^2+^, as described above. The collision cell was operated in the presence of a collision gas (N_2_), and the collision energy was set to 28 eV.

### Spiked test of NBM-derivatised peptides

Two spiked tests for the high concentration group (Bn-Cys and Bn-CGR) and low concentration group (Bn-CG and Bn-CGRT) were performed to assess the quantification performance of the calibration curve generated using standard solutions. A 10-fold diluted T34Q-X sample solution was spiked with the Bn-Cys and Bn-CGR standard at a final concentration of 0.2 μM. A 2-fold diluted T34Q-X sample solution was spiked with Bn-CG and Bn-CGRT standard at a final concentration of 0.02 μM. The recovery percentages were calculated by subtracting the amount detected in the non-spiked sample from the total amount.

### Analysis of the stability of HMCES-crosslinked DNA

To determine the half-life of the Q-GR adduct (a Gly-Arg peptide conjugated with the 4-carboxythiazolidine adduct; Figure 1A), 1 nM of total DNA in a mixture containing T34Q-GR (proteinase K-digested T34Q-HMCES) was 5′-^32^P labelled and dissolved in TE buffer (10 mM Tris–HCl pH 7.5, 1 mM EDTA) in the presence or absence of 55 nM of non-radiolabelled oligonucleotide C34G or T34G. Samples were incubated at 37°C and aliquots were collected at the indicated times. DNA was resolved on 10% polyacrylamide gels containing 7 M urea, and visualised and analysed using a Typhoon FLA 9000 (GE Healthcare).

For SDS-polyacrylamide gel electrophoresis (PAGE) analysis of crosslinked HMCES-ssDNA, samples were incubated with an equal volume of urea-SDS buffer (50 mM Tris–HCl pH 6.8, 10% glycerol, 4% SDS, 4 M urea, 570 mM β-mercaptoethanol) at 37°C for 10 minutes. To determine the stability of crosslinked HMCES-ssDNA, 20 nM of HMCES-crosslinked oligonucleotide T34Q-HMCES was incubated in buffer containing 20 mM HEPES–NaOH (pH 7.5), 50 mM NaCl, 0.2 mg/ml BSA, and 1 mM DTT in the presence or absence of 200 nM S10O, C34G, or T34G oligonucleotide. Reaction mixtures were incubated at 30°C for the indicated times and aliquots were collected. Samples were mixed with an equal volume of urea-SDS buffer and HMCES was visualised by western blot using an anti-HMCES antibody (ATLAS ANTIBODIES, Anti-HMCES HPA044968) and a Chemi-Lumi One L kit (Nacalai Tesque, 07880-70). Signals were detected and analysed using ImageQuant LAS 4000 Mini Biomolecular Imager (GE Healthcare).

### Primer extension

Annealing reactions were performed with 5′-^32^P-labelled primer and a 3.3-fold molar excess of template DNA at 4°C for 10 min. The reaction mixture (10 μl) contained 20 mM HEPES–NaOH (pH 7.5), 50 mM NaCl, 0.2 mg/ml BSA, 5 mM DTT, 5 mM MgCl_2_, 0.1 mM of each dNTP, 60 fmol primer annealed with 200 fmol template, and the indicated DNA polymerases. Proteins were combined on ice and reaction mixtures were incubated at 30°C for the indicated times. Reactions were terminated by the addition of 10 μl stop solution (30 mM EDTA, 94% formamide, 0.05% bromophenol blue, 0.05% xylene cyanole). Products were resolved on 10% or 8% polyacrylamide gels containing 7 M urea, and visualised and analysed using a Typhoon FLA 9000 (GE Healthcare).

### Endonuclease assays

AP endonuclease reactions were performed in a reaction buffer (10 μl) containing 20 mM HEPES–NaOH (pH 7.5), 50 mM NaCl, 0.2 mg/ml BSA, 5 mM DTT, and 10 mM MgCl_2_ supplemented with 60 fmol of 5′-^32^P labelled lesion-containing oligonucleotides annealed with 200 fmol of C34G, C31G+3, or C23G+11 oligonucleotide at 4°C for 10 min, and the indicated amounts of enzyme. For the nucleotide incision assays, 20 mM HEPES–NaOH (pH 7.5) and 10 mM MgCl_2_ were respectively replaced with 30 mM HEPES–NaOH (pH 6.8) and 0.1 mM MgCl_2_ as indicated. Reaction mixtures were incubated at 30°C for the indicated times. Reactions were terminated by the addition of 15 μl stop solution. Products were resolved on 20% polyacrylamide gels containing 7 M urea, and visualised and analysed using a Typhoon FLA 9000 (GE Healthcare). In reactions with APE2, MgCl_2_ was replaced in the reaction mixture with MnCl_2_ or CoCl_2_ at the indicated concentrations. DTT was omitted because DTT reduces Co^2+^. In the case of his-APE2(cat) assays, β-mercaptoethanol and glycerol were present at final concentrations of 2 mM and 2%, respectively, because they were introduced when volumetrically large amounts of enzyme preparation in storage buffer (50 mM HEPES–NaOH pH 7.5, 10 mM β-mercaptoethanol, 10% glycerol, and 300 mM NaCl) needed to be added to compensate for low activity of the enzyme preparations, i.e. the β-mercaptoethanol and glycerol came from the storage buffer. The concentrations of the other components, i.e. HEPES–NaOH and NaCl, were adjusted so that they arrived at the final concentrations indicated. In the case of FLAG-APE2 assays, the volume of the enzyme preparation added to the reaction represented 10% of the reaction volume; consequently, the reaction buffer contained 10% of the enzyme preparation buffer. Reactions were incubated at 30°C for 1 hour.

To prepare 3′-^32^P-labelled oligonucleotides, 100 pmol lesion-containing oligonucleotides were mixed with 340 pmol of a complementary oligonucleotide (C34G+3PG; Supplementary Table S1) in which the 3′-end was modified with phosphate to prevent degradation and extension by Klenow fragment and incubated at 4°C for 10 min for annealing. The 3′-ends of lesion-containing oligonucleotides were extended in reactions containing dGTP, dATP, dTTP and [α-^32^P]dCTP by Klenow fragment and then purified by ethanol precipitation. The resultant 3′-^32^P-labelled DNA was directly used in endonuclease reactions unless otherwise indicated. To make dsDNA with 3′-overhangs at both ends, the resultant DNA was mixed with a 100-fold molar excess of C31G+3 or C23G+11 oligonucleotide heated at 95°C for 30 s, and incubated at 4°C for 10 min. A marker DNA, T26G-C, was similarly prepared by 3′-labelling of the T26G oligonucleotide.

### TDP1 phosphodiesterase assay

The reactions were performed in a buffer (10 μl) containing 20 mM HEPES–NaOH (pH 7.5), 50 mM NaCl, 0.2 mg/ml BSA and 5 mM DTT supplemented with 60 fmol of the indicated lesion-containing oligonucleotides and the indicated amounts of enzyme. β-Mercaptoethanol and glycerol were present at final concentrations of 2 mM and 2%, respectively, which came from the enzyme storage buffer. There were no additional changes of the other components. Reaction mixtures were incubated at 30°C for 30 min. Reactions were terminated by the addition of 15 μl stop solution. Products were resolved on 20% polyacrylamide gels containing 7 M urea, and visualised and analysed using a Typhoon FLA 9000 (GE Healthcare).

## RESULTS

### Preparation of HMCES-crosslink- and 4-carboxythiazolidine adduct-containing oligonucleotides

HMCES reacts with AP sites in ssDNA (20,22–24). Crosslinking reactions were performed using recombinant HMCES with a histidine tag at the C-terminus (hereafter described as HMCES). Two oligonucleotides containing uracil residues (T70U and T34U) were used in this study (Figure 1A and Table S1). After crosslinking in the presence of UDG, HMCES-crosslinked to ssDNA was purified from the reaction mixture. First, remaining ssDNA and UDG were removed from the reaction mixture by Ni-affinity chromatography, and non-reacted HMCES was separated from ssDNA-crosslinked HMCES by MonoQ chromatography. Purified T70Q-HMCES and T34Q-HMCES were analysed by SDS-PAGE (Figure 1A–C). The gel was first stained with a fluorescence dye to detect DNA, and then stained with Coomassie Brilliant Blue (CBB). T70Q-HMCES and T34Q-HMCES were observed as slowly migrating forms and confirmed by DNA staining (Figure 1B and 1C). Fractions that migrated more slowly than T70Q-HMCES and T34Q-HMCES were detected that contained both DNA and protein (indicated by asterisks in Figure 1B and C), suggesting the presence of species of HMCES-crosslinked DNA that differed conformationally from the major product. We found that a significant amount of HMCES and T70O (an AP site containing oligonucleotide generated by UDG treatment of T70U, Figure 1A) co-purified with the crosslinked product (Figure 1B), possibly due to a high affinity between HMCES and ssDNA (20,22,23). The relative amount of contaminating T70O was calculated as 24% from the relative fluorescence signal intensities (Figure 1B) and as 33% from ^32^P-labelling of the 5′-OH of DNA (Supplementary Figure S1A and B). T34O (an AP site containing oligonucleotide generated by UDG treatment of T34U, Figure 1A) was less likely to co-purify with HMCES-crosslinked ssDNA (Figure 1C), with the relative amount of contaminating T34O calculated as 16% of the total DNA from 5′-^32^P -labelling of DNA (Supplementary Figure S1C and S1D).

To model protease digestion of crosslinked HMCES, a hyper-active mutant of HMCES, HMCES (W290G/L291A) (29), crosslinked to T34O, was digested with proteinase K at 37°C for 9 h. 5′-End labelling of the purified products with ^32^P and analysis by urea–PAGE revealed two major signals; one of which migrated with T34O. The other migrated more slowly (Figure 1D lane 3), suggesting that it represented peptide fragments conjugated with the 4-carboxythiazolidine adduct that had been incompletely digested by proteinase K (22). These proteinase K-digested T34Q-HMCES products are denoted as T34Q-X (X represents the remaining peptides with varying lengths).

### Determination of the peptide fragments remaining in the Q adduct

#### Derivatisation of cysteine with N-benzylmaleimide

To determine the peptide fragment of HMCES remaining in T34Q-X, T34Q-X was heated before LC–MS analysis, because 4-carboxythiazolidine adducts are heat labile (see the next section) (22). Since the N-terminal cysteine of HMCES reacts with the AP site (20,22,23), T34Q-X was expected to bear cysteine or short peptides containing cysteine. Thus, *N*-benzylmaleimide (NBM), which reacts with the sulfhydryl group in the cysteine side chain, was used to derivatise the peptides and allow their separation on a reversed-phase HPLC column and detection by UV absorption (Figure 1E). Maleimide has been used for site-specific modification of proteins (47), but there are only a few reports that describe its use for derivatisation of cysteine or biological thiols (48,49). Therefore, we analysed the reaction of NBM with cysteine first. When cysteine was incubated with NBM in water at 25°C for 24 h, in the presence of tris(carboxyethyl)phosphine hydrochloride (TCEP·HCl), a reducing agent that cleaves disulfide bonds, three product peaks were detected (Supplementary Figure S2A). The two peaks with retention times of 8.3 and 8.5 min exhibited the *m*/*z* value of the desired product (compound **3** X = OH in Figure 1E) in the LC–MS experiments. Since the Michael addition of a thiol to maleimide produces racemates, it is reasonable that two enantiomers were separated on the reversed-phase column. LC–MS experiments also revealed that the peak with a retention time of 6.4 min can be assigned to compound **4**, which is an intermediate in the reduction reaction of NBM with TCEP (50), When sodium phosphate buffer was used, an extra peak was detected at 14.8 min (Supplementary Figure S2B–S2G). The peaks of the derivatised cysteine and remaining NBM became smaller as the pH value of the solution increased. TCEP improved the yield of the desired products in all these experiments, and the derivatisation could be done effectively at pH 6.0 when NBM was present in excess (Supplementary Figure S2B). A peak of an unknown product also appeared when NBM was present in the buffer in the absence of the other reactants (Supplementary Figure S3B–D). Since the peak of this product was very small when the buffer was not used (Supplementary Figure S2A and S3A), it is possible that it resulted from the hydration or hydrolysis of maleimide. Co-injection experiments using the two expected products of maleimide hydration or hydrolysis, 1-benzyl-3-hydroxypyrrolidine-2,5-dione and *N*-benzylmaleamic acid, revealed that this peak was the latter (compound **5**), indicating that the peak was the result of hydrolysis of maleimide.

#### Derivatisation of peptide fragments of HMCES

After removal of low-molecular-weight compounds contaminating T34Q-X by gel filtration, the remaining peptide fragments of HMCES were cleaved by heating the solution in 10 mM HEPES–NaOH (pH 7.5) at 95°C for 10 min, and the oligonucleotide was removed by a second gel filtration. Since the retained fraction contained HEPES–NaOH, sodium phosphate at pH 6.0 was added in excess to the derivatisation reaction, together with TCEP·HCl. A large amount of NBM was used to complete derivatisation, as described in MATERIALS AND METHODS. As reference materials, cysteine and the N-terminal peptide fragments of HMCES including the dipeptide (CG), the tripeptide (CGR), and the tetrapeptide (CGRT) were derivatised without using sodium phosphate buffer (Supplementary Figure S4) (hereafter referred to as Bn-Cys, Bn-CG, Bn-CGR and Bn-CGRT, respectively). The thermostability of the peptides was confirmed by derivatising the compounds that had been heated at 95°C for 10 min in 10 mM HEPES–NaOH (pH 7.5). There was no difference between heated and unheated compounds (Supplementary Figure S5).

#### Quantification and identification of Bn-Cys, Bn-CG, Bn-CGR, and Bn-CGRT derivatised from T34Q-X

Supplementary Figure S6 shows the extracted ion chromatograms with 0.01 Da mass windows for Bn-Cys (*m/z* 309.091), Bn-CG (*m/z* 366.112), Bn-CGR (*m/z* 261.611), and Bn-CGRT (*m/z* 312.134) in standard solutions (Supplementary Figure S6A) and T34Q-X sample solution (Supplementary Figure S6B). Each NBM-derivatised peptide appeared as doublet peaks corresponding to enantiomers, similar to what was observed by HPLC-UV-VIS (Supplementary Figure S4). The differences in retention times between the T34Q-X sample and the derivatised compounds in the standard solutions were within 0.1 min. The *m/z* values of [M + H] ^+^ and [M + 2H] ^2+^ of the doublet peaks in the compounds in the standard solutions were in good agreement with the calculated value of each NBM-derivatised peptide. The results of the determination of the NBM-derivatised peptides in the T34Q-X sample including % coefficient of variation (CV), recovery percentages, limit of quantitation (LOQ; signal-to-noise ratio = 10) and observed *m/z* are shown in Table 1. The differences between the observed *m/z* values of [M + H] ^+^ and [M + 2H] ^2+^ in the T34Q-X sample and the calculated values of the NBM-derivatised peptides were <0.002 Da. One of the doublet peaks, which showed a good recovery percentage and a higher intensity than the other peaks, was adopted for the determination of the NBM-derivatised peptides. The calibration curves for Bn-Cys and Bn-CGR (five points of 0, 0.05, 0.1, 0.5 and 1.0 μM) or for Bn-CG and Bn-CGRT (five points of 0, 0.01, 0.02, 0.05 and 0.1 μM) were created by injecting the standard solution two times or more. The correlation coefficients of the calibration curves were satisfactory (>0.995). Recovery percentages were in agreement with the guidelines for standard method performance requirements (51,52), indicating that sample dilution and column separation successfully minimised background interference caused by residues of the biological matrix in the T34Q-X sample and the derivatising reagents. Therefore, the determination of the NBM-derivatised peptides was successfully carried out with calibration curves generated using standard solutions. As a result of the lower peak intensities of Bn-CG and Bn-CGRT, the repeatability of quantification of these compounds was not as good as that of Bn-Cys and Bn-CGR. However, it was considered that the amount of Bn-CG and Bn-CGRT in the T34Q-X sample was sufficient to allow a relative comparison with the higher amounts of Bn-Cys and Bn-CGR in the T34Q-X sample. The mass spectra of the NBM-derivatised peptides in the T34Q-X sample and the standard materials are shown in Supplementary Figure S7, and the product ion spectra of Bn-Cys and Bn-CGR obtained from MS/MS measurements are shown in Supplementary Figure S8. Several product ions were observed in the T34Q-X sample that had *m/z* values identical to those of the standard materials, and the relative intensities of the product ions in the T34Q-X sample were highly similar to those of the standard materials. The product ion spectra of Bn-CG and Bn-CGRT could not be obtained because of their low concentration. These results indicated that the T34Q-X sample contained cysteine (18.0%), CG (0.3%), CGR (81.5%) and CGRT (0.2%) (Table 1), suggesting that T34Q-X was a mixture of T34Q (4-carboxythiazolidine itself, Figure 1A) (18.0%), T34Q-G (T34Q containing one additional Gly residue) (0.3%), T34Q-GR (containing the additional Gly-Arg peptide, Figure 1A) (81.5%) and T34Q-GRT (containing the additional Gly-Arg-Thr peptide) (0.2%). Consequently, we estimated that, in the proteinase K-digested T34Q-HMCES (Figure 1D, lane 3) sample, the band migrating to a similar position to that of T34O is a mixture of T34O (approximately 7% of the products) and T34Q (approximately 17% of the products) and the band migrating slowly is T34Q-GR (approximately 76% of the products) (Figure 1D), based on the results of quantitative LC–MS analysis (Table 1) and the chemical properties of the T34Q-X oligonucleotides (see the next section).

**Table 1.** NBM derivatized peptides in the T34Q-X sample quantificated and identified by LC–MS analysis

NBM derivatized peptide	Retention time of the peak used for determination (min)	Measured amount in T34Q-X (pmol)	%CV	%Recovery (*n* = 2)	LOQ (pmol)	Observed *m/z*	Calculated *m/z*
Bn-Cys	16.9	162^a^	2	107	4	309.091^a^ [M + H]^+^	309.091
Bn-CG	17.7	2.8^b^	10	87	0.8	366.112^b^ [M + H]^+^	366.112
Bn-CGR	14.3	733^a^	1	92	4	261.609^a^ [M + 2H]^2+^	261.611
Bn-CGRT	14.6	1.5^b^	9	85	0.8	312.133^b^ [M + 2H]^2+^	312.134

^a^ *n* = 4.

^b^ *n* = 6.

### Factors affecting the stability of 4-carboxythiazolidine adducts

During the proteinase K treatment, we noticed that the relative amount of T34O gradually increased, suggesting instability of the 4-carboxythiazolidine adducts. We demonstrated that T34Q-GR was indeed heat labile at pH 7.5 and was converted to an AP site at 95°C regardless of the buffer used (Supplementary Figure S9A and S9B). The generation of AP sites was confirmed by examining the alkaline sensitivity of the heat-converted oligonucleotides, which was identical to that of the natural AP site containing oligonucleotide, T34O; both were cleaved by β-elimination followed by δ-elimination (Supplementary Figure S9C, compare lanes 4–6 with lanes 10–12, and Supplementary Figure S9D). By contrast, T34Q and T34Q-GR were relatively alkaline resistant (Supplementary Figure S9C, compare lanes 1–3 with lanes 7–9). These results demonstrate that 4-carboxythiazolidine adducts are heat labile, irrespective of whether or not they are conjugated with short peptides. The half-life of the Q-GR adduct at 37°C and pH 7.5 was 24 hours (Figure 1F and G). Surprisingly, the half-life of the Q-GR adduct decreased to 12 hours in the presence of an excess amount of a complementary ssDNA, C34G, but only to 20 hours in the presence of a non-complementary ssDNA, T34G (Figure 1F and G), suggesting that the Q-GR adduct was destabilised in double-stranded DNA (dsDNA).

The cross-linkage between HMCES and ssDNA is stable (22), although it is reversible (24). However, it is uncertain whether it is stable in dsDNA. We confirmed the stability of the HMCES-ssDNA crosslink and that the quantity of T34Q-HMCES remained constant throughout a 60 min incubation in the absence of any additional DNA or presence of excess S10O, used to trap de-crosslinked HMCES (Figure 1H and I) (24). When T34Q-HMCES was incubated in the presence of the complementary ssDNA C34G, but not when it was incubated in the presence of non-complementary ssDNA T34G, we found that the crosslink was unstable (Figure 1H and I), resulting in an apparent half-life of approximately 3.5 h. These results suggest that the de-crosslinking reaction is stimulated by the formation of dsDNA.

Since the high affinity of HMCES for ssDNA and the instability of 4-carboxythiazolidine adducts indicated that it would be difficult to purify further the constructs, the above-described DNA constructs (Figure 1B, C, lane 2 and D, lane 3) were used in downstream experiments without further purification. It was confirmed that the spontaneous cleavage of contaminating T34O by β-elimination was negligible in the final preparations (Figure 6B–E, lanes without enzyme), indicating that the AP site-containing DNAs, T70O and T34O, remained intact during preparation as shown schematically in Figure 1A.

### Crosslinking of HMCES to AP sites blocks DNA synthesis

To prepare primer-templates for primer extension experiments, we omitted the pre-heat treatment step and the subsequent slow-cooling step for annealing because of the heat instability of HMCES-crosslink- and Q-adducts. For efficient annealing, we added a 3.3-fold molar excess of template to primer and incubated at 4°C for 10 min. Since almost all primers annealed to templates under this condition, we used this annealing condition in the following experiments. However, some primers did not anneal efficiently to a HMCES-crosslinked oligonucleotide (see section ‘TLS through the HMCES-crosslink and 4-carboxythiazolidine adducts’). To accommodate for this, we determined the amount of primer that annealed to the template to evaluate the efficiency of the primer extension reactions. Efficient polymerase activity was clearly detected even in the presence of the excess ssDNA, as demonstrated in the respective control experiments (see below).

**Figure 2.**
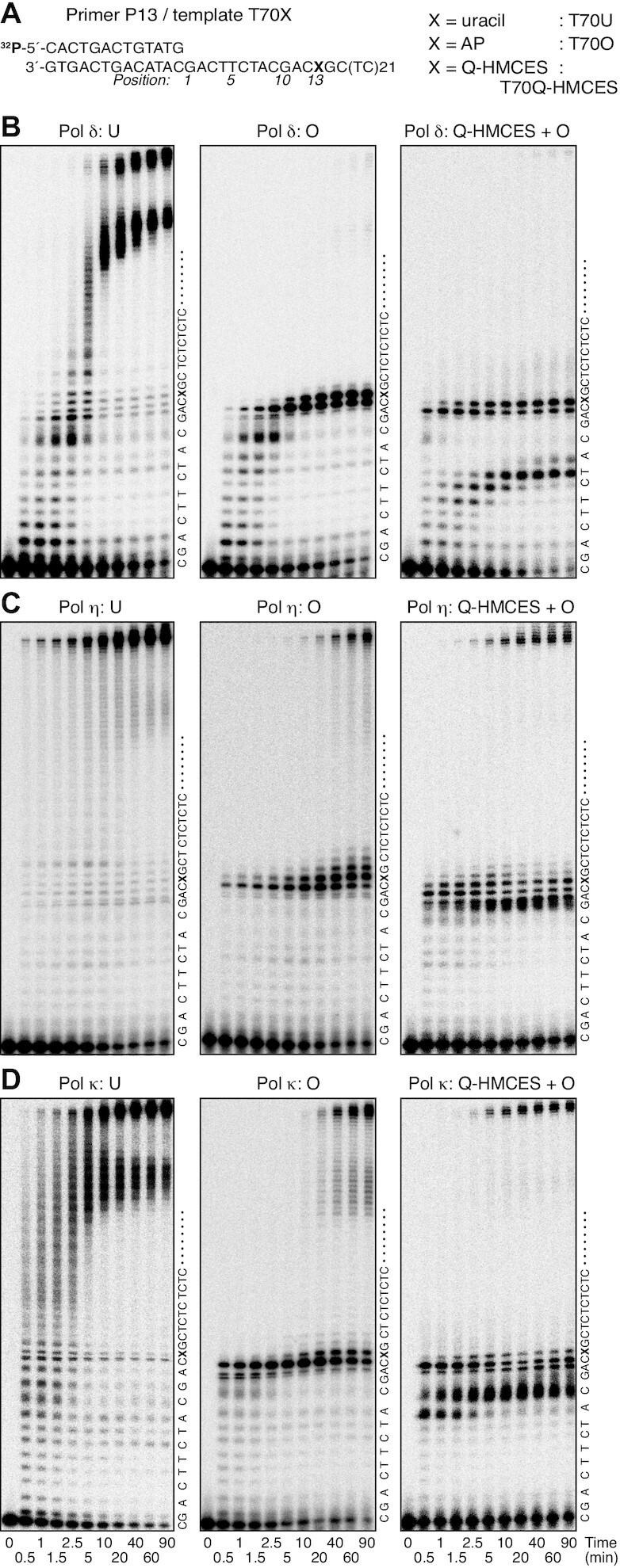
Primer extension assay with HMCES-crosslink-containing templates. (**A**) Nucleotide sequences of the 5′-^32^P-labelled primer and templates used in the assays. The assays containing the T70Q-HMCES template were performed using a template mixture of T70Q-HMCES (67%) and T70O (33%), as shown in Supplementary Figure S1B. (**B–D**) Time courses of primer extension reactions. Pol δ (35 fmol; B), pol η (35 fmol; C), or pol κ (70 fmol; D) was incubated with the indicated primer-template at 30°C for the indicated times. The reaction products were resolved by 10% urea–PAGE. The nucleotide sequences of the template strands are shown to the right of each panel.

Primer extension reactions were performed with a replicative DNA polymerase, pol δ, using T70-HMCES as a template and T70U and T70O as template controls (Figure 2A and B). The majority of DNA synthesis passed through uracil in the T70U control after 10 min (Figure 2B, left panel). With template T70O, the amount of the product arrested at position 12 increased during the first 5 min of the reaction, but then gradually decreased with further incubation. Concomitantly, the amount of the product arrested at position 13 started to increase (Figure 2B, middle panel). This result indicates that pol δ paused one base upstream of the AP site before stalling opposite the template AP site after one base insertion. Time course reactions with a template mixture (T70Q-HMCES and T70O) revealed two major pausing sites (Figure 2B, right panel); positions 12 and 13, and position 7 (Figure 2B, right panel). Since positions 12 and 13 were the pausing sites at the AP site on template T70O (Figure 2B, middle panel), we considered position 7 as the site where pausing occurred as a result of HMCES-crosslinking on the T70Q-HMCES template. This result demonstrated that HMCES crosslinked to ssDNA inhibited DNA replication by pol δ at the 6 bases upstream of the crosslinking site. Kinetic analysis further revealed that the product pausing at position 12 first accumulated for 2.5 min, decreased for 5–10 min, and then increased until 90 min (Figure 2B, right panel), suggesting that a small fraction of the product that stalled at 6 bases upstream of the crosslinking site was extended by at least one base upstream of the crosslinking site during long incubation (Figure 2B, right panel).

Next, primer extension reactions with the translesion polymerases pol η and pol κ were performed (Figure 2C and D). The majority of DNA synthesis by pol η and pol κ passed through uracil in T70U (Figure 2C and D, left panels). With template T70O, pol η paused at position 12, gradually progressed to position 13, before elongating further after a long incubation (Figure 2C middle panel). A significant fraction of the reactions reached the end of the template, suggesting bypass of the AP site (Figure 2C, middle panel). Replication of a template mixture (T70Q-HMCES and T70O) by pol η revealed pausing at positions 10–13 (Figure 2C right panel). Since positions 12 and 13 were the pausing sites at the AP site on template T70O (Figure 2C, middle panel), positions 10 and 11 were regarded as the sites of pausing caused by HMCES-crosslinking on the T70Q-HMCES template (Figure 2C, right panel). These data demonstrated that the HMCES-ssDNA crosslink inhibited DNA replication by pol η at 2–3 bases upstream of the crosslinked site. Furthermore, reaction kinetics, again, revealed that the products at positions 12 and 13 first increased for 2.5 min, decreased for 20 min, and then increased until 90 min, with a concomitant decrease in the pausing product at positions 10 and 11 (Figure 2C, right panel), suggesting that a small fraction of reactions that had stalled upstream of the crosslink were able to extend opposite the crosslinking site.

Pol κ paused at positions 10–12 when incubated with T70O, before gradually extending to position 13 (Figure 2D, middle panel). A significant fraction of reactions reached the end of the template (Figure 2D middle panel), suggesting bypass of the AP site. DNA synthesis with the template mixture (T70Q-HMCES and T70O) revealed two pausing sites at positions 8 and 9 and 11–13 (Figure 2D right panel). Since positions 11–13 were the pausing sites at the AP site of template T70O (Figure 2D, middle panel), positions 8 and 9 were regarded as the sites where pausing occurred due to HMCES-crosslinking on the template T70Q-HMCES. This result demonstrated that HMCES-ssDNA crosslinks inhibit DNA replication by pol κ at 4–5 bases upstream of the crosslinking site. Furthermore, the kinetics of the reaction also revealed that the products pausing at positions 12 and13 first accumulated for 2.5 min, decreased for 20 min, and again increased until 90 min, with a concomitant decrease of the pausing product at position 9 (Figure 2D, right panel), suggesting that a small fraction of the products that stalled at 4 bases upstream of the crosslinking site were extended at least opposite the crosslinking site.

**Figure 3.**
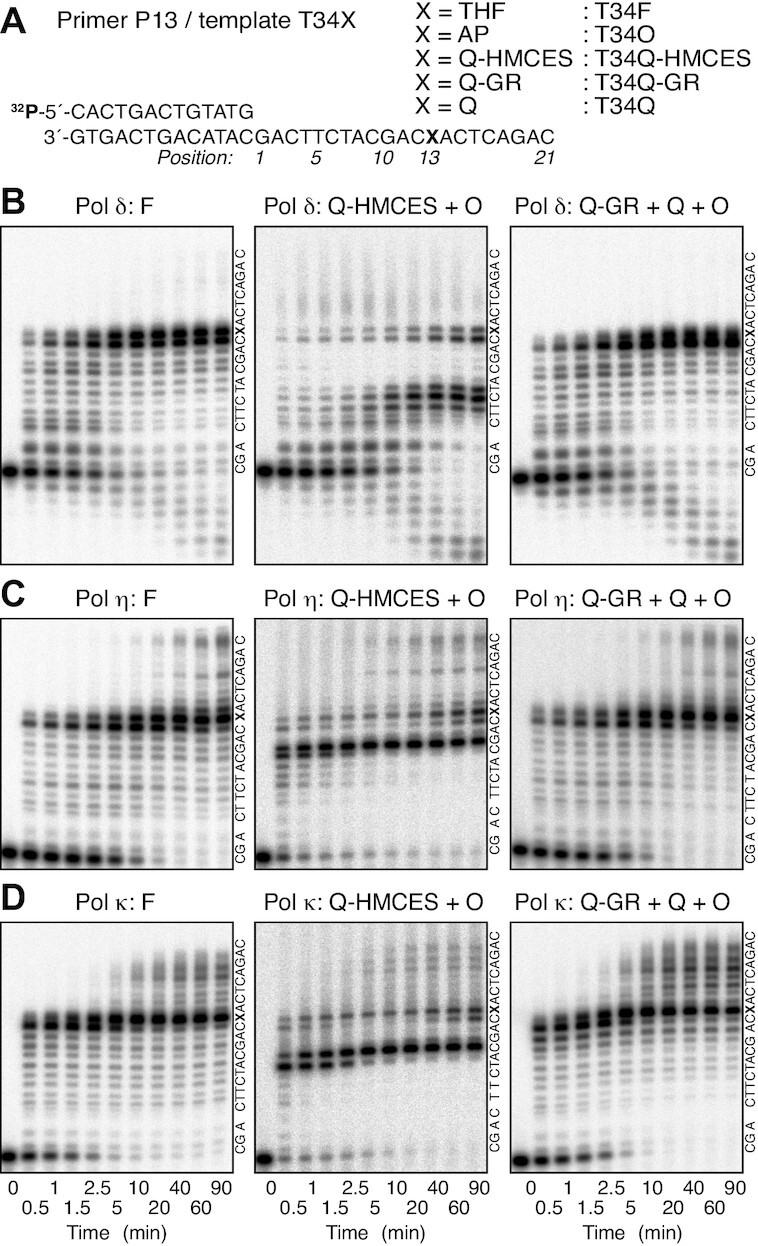
Primer extension with templates containing HMCES-crosslink or 4-carboxythiazolidine adducts. (**A**) Nucleotide sequences of the 5′-^32^P-labelled primer and templates used in the assays. The assays containing the T34Q-HMCES template were performed using a template mixture of T34Q-HMCES (84%) and T34O (16%), as shown in Supplementary Figure S1D, and those containing the T34Q-GR template were performed using a template mixture of T34Q-GR (76%), T34Q (17%), and T34O (7%), as shown in Figure 1D, lane 3. (**B–D**). Time courses of primer extension reactions. Pol δ (35 fmol; B), pol η (35 fmol; C), or pol κ (70 fmol; D) was incubated with the indicated primer-template at 30°C for the indicated times. The reaction products were resolved by 8% urea–PAGE. The nucleotides sequences of the template strands are shown to the right of each panel.

Next, T34Q-HMCES was examined as a template (Figure 3). We expected that the effects of HMCES-ssDNA crosslinks would be more clearly observed with the T34Q-HMCES template than with the T70Q-HMCES template because the amount of the contaminating AP site-containing template was smaller in T34Q-HMCES (Supplementary Figure S1). Note that the nucleotide sequence at the 3′ side of the crosslinking site is identical to that in T70Q-HMCES, while the nucleotide sequence at the 5′ side differs (Figures 2A and 3A). A THF containing template, T34F, was used as a control for TLS through the AP site (Figure 3A), because natural AP site-containing templates could contain a trace amount of uracil-containing template that had not been completely converted by UDG treatment. However, it should be noted that polymerases do not respond to THF sites exactly in the same way as they do to natural AP site (53).

The time course for primer extension with pol δ is shown in Figure 3B. With template T34F, the polymerase stalled at positions 12 and13 and the products accumulated for 5 min (Figure 3B, left panel). A significant fraction of degraded primer was visible, which was likely due to the exonuclease activity of pol δ (Figure 3B left panel). This result indicates that DNA synthesis with pol δ pauses one base in front, or opposite the template THF. Reactions with a template mixture (T34Q-HMCES and T34O) revealed two major pausing sites: at positions 5–7 and 12 and 13 (Figure 3B, middle panel). As expected, the fractions that accumulated at positions 12 and13 at early time points, which correspond to the pausing sites at the AP site on T34O, were smaller and the fractions that accumulated at positions 5–7, which corresponded to the pausing sites caused by HMCES crosslinked to ssDNA on the T34Q-HMCES template, were larger than those that accumulated with the template mixture containing T70Q-HMCES (Figure 2B right panel). At later time points, further accumulation at positions 12 and13 was observed (Figure 3B, middle panel). These results suggest that HMCES crosslinked to ssDNA inhibits pol δ at 6–8 bases upstream of the crosslink, but that the polymerase could progress at least to the crosslinking site (Figure 3B, middle panel).

Extension by the translesion DNA polymerases pol η and pol κ were similarly analysed (Figure 3C and D). With templates T34F, pol η and pol κ paused at position 12 and gradually extended to position 13, while a small fraction of primers was extended beyond the lesion after 5 min incubation. The pausing at the position 12 was more persistent with pol η than with pol κ (Figure 3C and D, left panels). When a template mixture (T34Q-HMCES and T34O) was examined, pol η paused at positions 9 and10 and positions 12 and 13 (Figure 3C, middle panel). Positions 9 and 10 were regarded as the sites where pausing occurred due to HMCES-crosslinking on the template T34Q-HMCES. The fraction that paused at position 12 decreased during 5–10 min of incubation and then increased, with concomitant accumulation at position 13 (Figure 3C middle panel). These results demonstrated that HMCES crosslinked to ssDNA inhibits DNA replication by pol η 3–4 bases upstream of the crosslink in the majority of cases, while a small fraction of primers was extended to a position opposite the crosslinking site (Figure 3C middle panel). Pol κ paused at positions 8 and 9 and positions 12 and 13 on the templates in the template mixture (T34Q-HMCES and T34O) (Figure 3D, middle panel). Positions 8 and 9 were regarded as the sites where pausing occurred due to HMCES-crosslinking on the T34Q-HMCES template. The accumulation at positions 12 and 13 at later time points (Figure 3D, middle panel) was similar to what was observed with pol η (Figure 3C middle panel). These results demonstrate that HMCES crosslinked to ssDNA inhibits DNA replication by pol κ 4–5 bases upstream of the crosslink site, while a small fraction of primers was extended to a position opposite the crosslinking site. These data are essentially identical to those obtained with the T70-HMCES template (Figure 2). We confirmed that the HMCES-crosslink was stable during primer extension reactions, indicating that crosslink reversal was not induced as a replication intermediate (Supplementary Figure S10).

### 4-Carboxythiazolidine adducts inhibit DNA synthesis

It is suggested that crosslinked HMCES is degraded by the proteasome *in vivo* (20). Thus, we examined TLS of proteinase K-digested T34Q-HMCES adducts as a model (Figure 1D lane 3). When the template mixture (T34Q-GR, T34O and T34Q) was used as a template for pol δ, pausing at positions 5–7 by T34Q-HMCES did not occur, while pausing at positions 12 and 13 persisted (Figure 3B, right panel). The overall kinetics observed with the template mixture were quite similar to those of the T34F template (Figure 3B, compare between right and left panels). These results suggest that 4-carboxythiazolidine adducts inhibit pol δ similarly to THF. When the template mixture (T34Q-GR, T34O and T34Q) was incubated with pol η, no pausing at positions 9 and 10 was observed, while pausing at positions 12 and 13 increased (Figure 3C right panel). The overall kinetics of replication observed with the template mixture were quite similar to those of the T34F template (Figure 3C, compare between right and left panels). These results suggest that 4-carboxythiazolidine adducts inhibit pol η similarly to THF. When the mixed template was incubated with pol κ, the pausing sites at positions 8 and 9 observed with T34Q-HMCES were no longer observed, while pausing at positions 11–13 increased (Figure 3D, right panel). The overall kinetics of DNA replication with the template mixture were again quite similar to those of the T34F template (Figure 3D, compare between right and left panels). These results suggest that 4-carboxythiazolidine adducts inhibit pol κ similarly to THF.

Lastly, we examined the activity of pol β, a repair DNA polymerase. We confirmed that pol β stalled one base upstream of an THF site and no incorporation occurred opposite the template THF, as reported previously (53). Similarly, no incorporation was observed opposite template 4-carboxythiazolidine adducts. The HMCES-crosslink also completely blocked DNA synthesis by pol β (Supplementary Figure S11).

### TLS through the HMCES-crosslink and 4-carboxythiazolidine adducts

**Figure 4.**
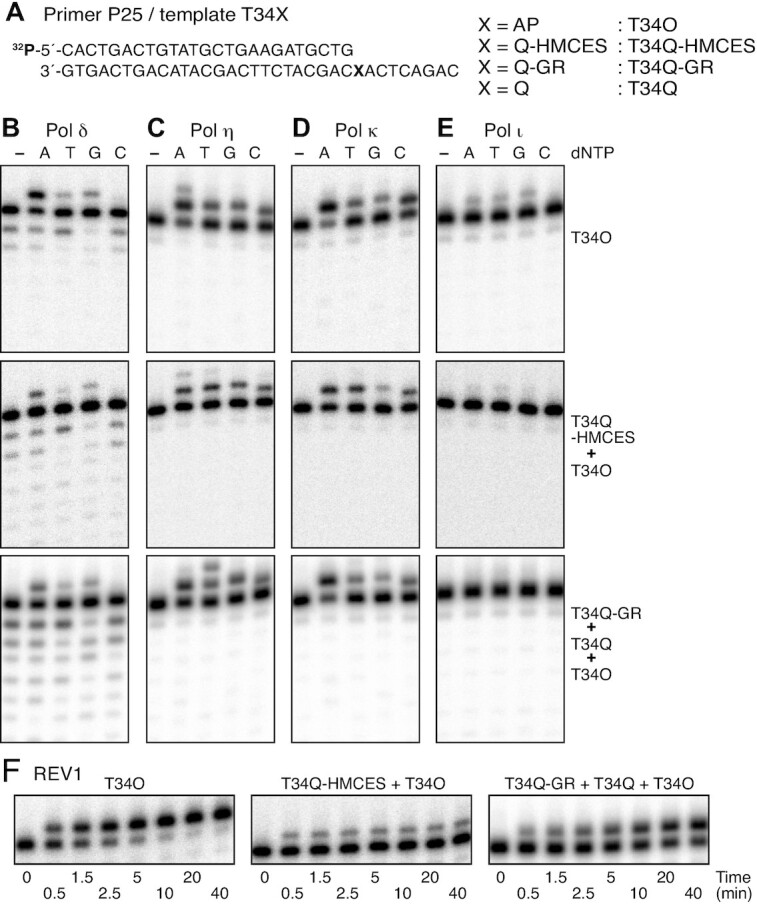
Nucleotide incorporation opposite HMCES-crosslink or 4-carboxythiazolidine adducts. (**A**) Nucleotide sequences of the 5′-^32^P-labelled primer and templates used in the assays. The assays containing the T34Q-HMCES template were performed using a template mixture of T34Q-HMCES (84%) and T34O (16%), as shown in Supplementary Figure S1D, and those containing the T34Q-GR template were performed using a template mixture of T34Q-GR (76%), T34Q (17%), and T34O (7%), as shown in Figure 1D, lane 3. (**B–E**) Reactions were performed in the presence of one dNTP. Pol δ (35 fmol; B), pol η (35 fmol; C), pol κ (70 fmol; D) or pol ι (74 fmol; E) was incubated with the indicated primer-template and dNTP at 30°C for 10 min. The reaction products were resolved by 8% urea–PAGE. (**F**) Time courses of the dCMP transferase activity of REV1. REV1 (43 fmol) was incubated with the indicated primer-template and dCTP at 30°C for the indicated times. The reaction products were resolved by 8% urea–PAGE.

The HMCES-crosslink and 4-carboxythiazolidine adducts blocked DNA synthesis by both replicative and Y-family TLS polymerases; however, inefficient nucleotide insertion was observed. Next, we examined the nucleotides inserted opposite the HMCES-crosslink and 4-carboxythiazolidine adducts. A 5′-^32^P-labelled oligonucleotide, P25, was annealed to the templates in the template mixtures (T34Q-HMCES and T34O) and (T34Q-GR, T34Q and T34O), or to the T34O template alone, and the extension products were examined following DNA synthesis in the presence of individual nucleotides (Figure 4A). Note that the HMCES-crosslink partially inhibited the annealing reaction and the annealed products appeared to be unstable even in the presence of 3.3-fold molar excess of templates, although the excess amount of templates improved the annealing reaction (Supplementary Figure S12). We used the same annealing condition as described above for the following primer extension experiments, although a significant fraction of primer was not annealed. Under the assay conditions, 52% and 26% of the primer annealed to the T34Q-HMCES template and the T34O template in the mixture, respectively, and 22% remained unannealed (Supplementary Figure S12).

Pol δ inserted dAMP preferentially when the primer was annealed to T34O and to the templates in the template mixtures (T34Q-HMCES and T34O) and (T34Q-GR, T34Q and T34O) (Figure 4B). Pol η and pol κ similarly inserted dAMP preferentially opposite the lesions in the T34O template and in the template mixture (T34Q-GR, T34Q and T34O) (Figure 4C and D, top and bottom panels), while such preferential insertion of dAMP was diminished in the template mixture (T34Q-HMCES and T34O) (Figure 4C and D, middle panels). The response of pol κ to natural AP sites was consistent with that reported previously (53). Pol ι inserted dAMP, dTMP, and dGMP inefficiently opposite the AP sites (Figure 4E, top panel), and failed to extend primers annealed to the template mixtures containing T34Q-HMCES or T34Q-GR (Figure 4E middle and bottom panels). REV1 efficiently inserted dCMP opposite template AP sites (54–56). The kinetic experiment demonstrated that the majority of the primers were extended after a 10 min incubation (Figure 4F left panel and Supplementary Figure S13). The insertion of dCMP by REV1 in the template mixture (T34Q-GR, T34Q and T34O) was inefficient (Figure 4F, right panel). The amount of the extended primers gradually increased during a 40 min incubation, representing extension of up to 60% of the total primers added to the reaction (Figure 4F, right panel and Supplementary Figure S13). This indicated that the Q-GR adduct is not as good a substrate for REV1 as the AP site. With the template mixture (T34Q-HMCES and T34O), the reactions were saturated after a 5 min incubation, during which ∼30% of the total primers were extended (Figure 4F middle panel and Supplementary Figure S13). The level nearly corresponded to 26% of the primers annealed to the T34O template in the template mixture (Supplementary Figure S12). This result suggests that only the primers annealed to T34O were extended and thus REV1 hardly inserts dCMP opposite the HMCES-crosslink.

**Figure 5.**
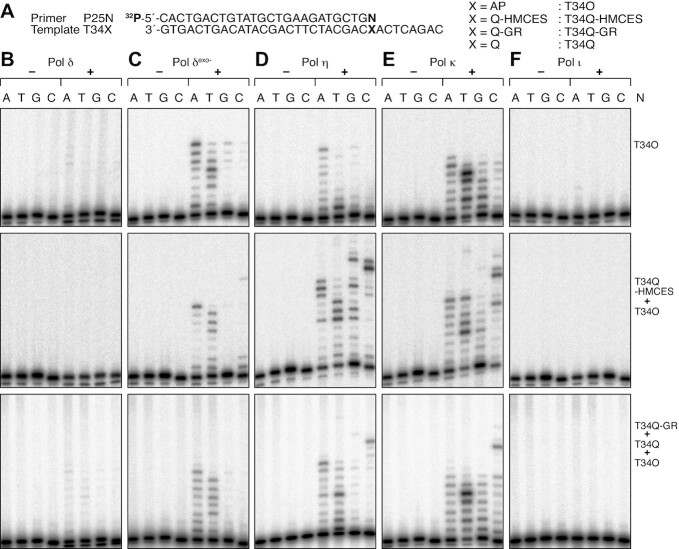
Primer extension assays beyond the HMCES-crosslink and 4-carboxythiazolidine adducts. (**A**) Nucleotide sequences of the 5′-^32^P-labelled primers and templates used in the assays. N represents A, T, G, or C. The assays containing the T34Q-HMCES template were performed using a template mixture of T34Q-HMCES (84%) and T34O (16%), as shown in Supplementary Figure S1D, and those containing the T34Q-GR template were performed using a template mixture of T34Q-GR (76%), T34Q (17%), and T34O (7%), as shown in Figure 1D, lane 3. (**B–F**). Reactions were performed with the indicated primer-templates. Pol δ (35 fmol; B), pol δ exo^−^ (75 fmol; C), pol η (35 fmol; D), pol κ (70 fmol; E), or pol ι (74 fmol; F) was incubated at 30°C for 10 min. The reaction products were resolved by 8% urea–PAGE.

Next, we examined the extension step beyond the HMCES-crosslink and 4-carboxythiazolidine adducts by annealing four primers with different 3′-terminal nucleotides that mimic the insertion of A, T, G and C opposite the lesion (labelled N in Figure 5A). Pol δ hardly extended these annealed primers in the presence of any tested lesion (Figure 5B), suggesting that the HMCES-crosslink and 4-carboxythiazolidine adducts strongly inhibited extension through the lesion. This inhibition could be due, in part, to the proofreading activity of pol δ. Indeed, an exonuclease defective mutant of pol δ, polδ^exo−^, clearly extended primers P25A and P25T but less clearly primers P25G and P25C with template T34O (Figure 5C, top panel). With the template mixture (T34Q-HMCES and T34O) or (T34Q-GR, T34Q and T34O), the extension reactions were more inefficient than with the template T34O (Figure 5C, middle and bottom panels). Interestingly, a faint product was detected following extension of P25C that was longer than the template strand with the template mixture (T34Q-HMCES and T34O) (Figure 5C, middle panel), suggesting that the polymerase could be reading back via template slippage in front of the adduct. Moreover, the final extension product from P25T with the template mixture (T34Q-GR, T34Q and T34O) was one base shorter than that from P25A (Figure 5C, bottom panel), suggesting template slippage, during which the 3′-thymine of the primer may anneal with the adenine of the template located next to the lesion (Figure 5A).

Pol η extended most efficiently from P25A with template T34O (Figure 5D top panel), while pol κ extended from P25A, P25T and P25G, but inefficiently from P25C (Figure 5E top panel). Surprisingly, with the template mixture (T34Q-HMCES and T34O), pol η and pol κ generated products longer than the template strand from all primers (Figure 5D and E, middle panels). With the template mixture (T34Q-GR, T34Q and T34O), pol η generated a product that was one base longer than the template sequence from P25A and P25T, and much longer products from P25G and P25C (Figure 5D, bottom panel). When the template mixture (T34Q-GR, T34Q and T34O) was incubated with pol κ, the longest products extended from P25A, P25T and P25G were at least one base longer than those detected in reactions with T34O, and much longer products were also detected in reactions with P25C (Figure 5E, bottom panel). Pol ι was unable to extend any primer annealed to any damaged template. Together, these data indicate that the HMCES-crosslink is a strong replication-blocking adduct and that the Q-GR adduct inhibits replication to a similar extent as the AP site, although it seems to make TLS DNA polymerases prone to template slippage.

### The activity of nucleases on 4-carboxythiazolidine adducts

**Figure 6.**
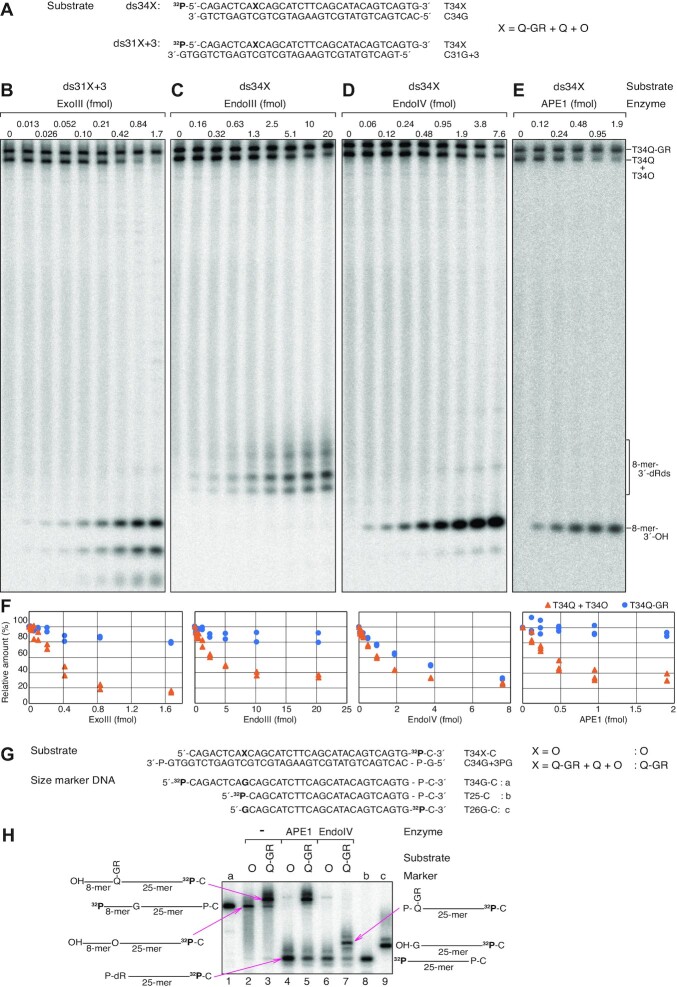
Endonuclease assays of 4-carboxythiazolidine adducts under the AP endonuclease reaction condition. (**A**) Nucleotide sequences of 5′-^32^P-labelled substrates used in B-E. An oligonucleotide mixture of T34Q-GR (50%), T34Q (10%), and T34O (40%) was annealed with C34G and C31G+3 to generate the substrates designated as ‘ds34X’ and ‘ds31X+3’, respectively. (**B–E**) The indicated substrates were incubated with the indicated enzymes at 30°C for 10 min under the 10 mM MgCl_2_ at pH 7.5 reaction condition. Reaction products were resolved by 20% urea–PAGE. The product of incision 5′ to the lesions is indicated as ‘8-mer-3′-OH’. The products of β-elimination at the AP site are indicated as ‘8-mer-3′-dRds’, with dRds representing deoxyribose derivatives. (**F**) The relative radioactivity of T34Q-GR and T34Q + T34O measured in the gel images of three (APE1) or two (ExoIII, EndoIII, and EndoIV) independent experiments, as described in B–E. (**G**) Nucleotide sequences of substrates and marker oligonucleotides used in (H). Radioactive labelling is indicated. T34O and a mixture of T34Q-GR (76%), T34Q (17%), and T34O (7%) were 3′-labelled with [α-^32^P]dCTP to generate the substrates designated as ‘O’ and ‘Q-GR’, respectively. (**H**) Endonuclease assays of APE1 and EndoIV with 3′-^32^P-labelled substrates. The indicated substrates were incubated with APE1 (0.2 pmol) or EndoIV (6.3 fmol) at 30°C for 1 h under the 10 mM MgCl_2_ at pH 7.5 reaction condition. The reaction products were resolved by 20% urea–PAGE.

Next, we examined whether 4-carboxythiazolidine adducts are repaired by the BER pathway. An approximately 5:1:4 mixture of T34Q-GR, T34Q, and T34O were 5′ labelled with ^32^P and annealed to the complementary ssDNA C34G or C31G+3, in which cytosine is located opposite the lesions (Figure 6A). dsDNA generated following annealing to C34G (designated as substrate ‘ds34X’) was blunt ended, while dsDNA generated following annealing to C31G+3 (designated as substrate ‘ds31X+3’) had 3-base 3′-overhangs at both ends of the dsDNA, prohibiting exonuclease activity (Figure 6A). To make dsDNA, a 3.3-fold molar excess of the complementary strand was added to induce efficient annealing of all the lesion-containing oligonucleotides without the need for pre-heat treatment and subsequent slow-cooling. Almost all the lesion-containing oligonucleotides in the mixture annealed to the complementary strand after incubation at 4°C for 10 min. Efficient incision activity was clearly detected even in the presence of excess ssDNA in the respective control experiments (see below).

We first examined the activity of well characterised *E. coli* AP endonucleases (ExoIII and EndoIV) (57). We also tested an AP lyase (EndoIII) as a control to confirm that 4-carboxythiazolidine adducts are indeed resistant to the lyase reaction mediated by the β-elimination. The reactions were performed in a reaction containing 10 mM MgCl_2_ at pH 7.5, a condition under which the AP endonuclease activities of ExoIII (58) and EndoIV (59–61) were efficiently detected previously. ExoIII and EndoIII cleaved the fraction containing T34Q + T34O (Figure 6B, C and F). By contrast, EndoIV cleaved both fractions containing T34Q + T34O and T34Q-GR with similar efficiency (Figure 6D and F), indicating that EndoIV can initiate repair of 4-carboxythiazolidine adducts in *E. coli*. We also examined the activity of cell lysates produced from the ExoIII/EndoIV-deficient and -proficient isogenic *E. coli* strains, RPC501 and AB1157, respectively (40) (Supplementary Figure S14). Lysates from the nuclease proficient strain AB1157 exhibited cleavage activity, as demonstrated by the generation of an 8-mer-3′-OH product. This activity was abolished in the ExoIII/EndoIV-deficient lysate from RPC501 (Supplementary Figure S14).

Next, we examined the activity of the human AP endonuclease, APE1, under a reaction containing 10 mM MgCl_2_ at pH 7.5, a condition under which the AP endonuclease activity of APE1 was efficiently detected previously (39,62–65). A recombinant APE1 had little activity on the Q-GR adduct (Figure 6E and F) even in the presence of a large excess of the enzyme (up to 13 pmol) during long incubation (60 min) (Supplementary Figure S15B and S15C) whereas it exhibited robust activity on AP sites with as little as 1 fmol APE1 after a 10 min incubation (Figure 6E and F).

To confirm incision by EndoIV, we incubated the protein with 3′-^32^P-labelled substates generated by the addition of [α-^32^P]dCMP and examined the reaction products (Figure 6G). As a control, excess APE1 or EndoIV was incubated with a 3′-labelled substrate derived from T34O (designated as substrate ‘O’ in Figure 6G and H), generating a product containing a 5′-phosphodeoxyribose (5′-P-dR) moiety (Figure 6H, lanes 4 and 6) (57,66,67). When excess APE1 was incubated with the 3′-labelled substate mixture derived from T34Q-GR, T34Q and T34O (designated as substrate ‘Q-GR’ in Figure 6G and H), 3′-labelled T34O was cleaved but 3′-labelled T34Q-GR was not (Figure 6H, compare lanes 3 and 5), indicating that 3′-labelled T34Q-GR was resistant to APE1 under this assay condition. By contrast, when EndoIV was incubated with this mixture, all substrates were cleaved and bands corresponding to two major products (a band corresponding to a cleaved AP site and a band migrating more slowly than the T26G-C marker) appeared (Figure 6H lanes 7 and 9), suggesting cleavage of 3′-labelled T34Q-GR. These results suggest that EndoIV incises DNA 5′ to the Q-GR adduct, generating a 3′-OH and a P-Q-GR moiety at the 5′-end.

**Figure 7.**
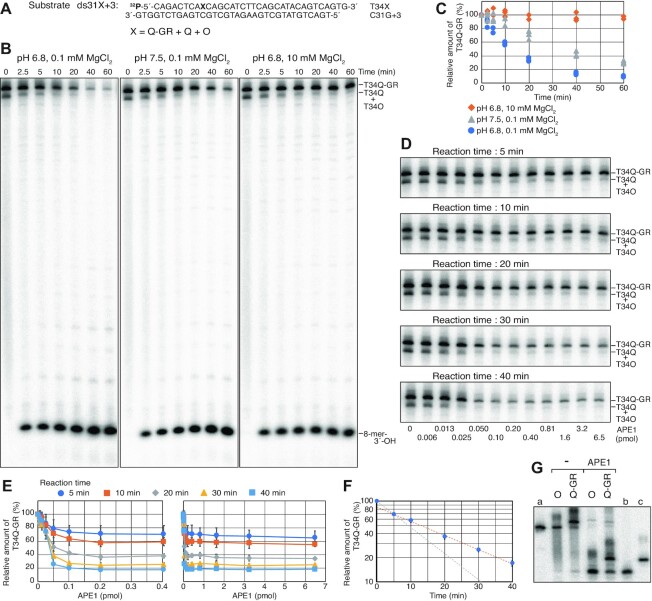
Incision assays of 4-carboxythiazolidine adducts under the nucleotide incision reaction condition. (**A**) Nucleotide sequences of the 5′-^32^P-labelled substrates used in B and D. An oligonucleotide mixture of T34Q-GR (76%), T34Q (17%), and T34O (7%) was annealed with C31G+3 to generate the substrates designated as ‘ds31X+3’. (**B**) The substrate ds31X+3 was incubated with 0.1 pmol APE1 at 30°C for the indicated times under the indicated reaction conditions. Reaction products were resolved by 20% urea–PAGE. The product of incision 5′ to the lesions is indicated as ‘8-mer-3′-OH’. (**C**) The relative radioactivity of T34Q-GR measured in the gel images of three independent experiments, as described in (B). (**D**) The substrate ds31X+3 was incubated with the indicated amounts of APE1 at 30°C for the indicated times under the 0.1 mM MgCl_2_ at pH 6.8 reaction condition. Reaction products were resolved by 20% urea–PAGE. The untrimmed images are shown in Supplementary Figure S16. (**E**) The relative radioactivity of T34Q-GR was measured in the gel images of seven (5 min), four (10 min) or three (20, 30 and 40 min) independent experiments, as described in D. Averages were plotted with SD in a range of 0–0.4 pmol (left) and 0–6.5 pmol (right) APE1. (**F**) Averages of the relative amounts of the remaining T34Q-GR in the reactions in the presence of saturating amounts of APE1 (0.2, 0.4, 0.81, 1.6, 3.2 and 6.5 pmol) in E were plotted against the incubation times with SD. (**G**) Incision assays of APE1 with 3′-^32^P-labelled substrates. The indicated substrates described in Figure 6G were incubated with APE1 (0.1 pmol) at 30°C for 40 min under the 0.1 mM MgCl_2_ at pH 6.8 reaction condition. The reaction products were resolved by 20% urea–PAGE. The size markers, a, b and c are shown in Figure 6G.

Since APE1 is known to cleave DNA 5′ to some oxidised bases under acidic and low Mg^2+^ conditions, referred to as nucleotide incision activity (62,63,68–71), we tested whether APE1 can incise the Q-GR adduct under these assay conditions. As shown in Figure 7, APE1 clearly exhibited incision activity on the Q-GR adduct in reactions containing 0.1 mM MgCl_2_ at pH 6.8 (Figure 7B and C). In reactions containing 0.1 mM MgCl_2_ at pH 7.5 incision activity was reduced, and in reactions containing 10 mM MgCl_2_ at pH 6.8 (Figure 7B and C) incision activity was no longer observed. These results indicated that both low pH and Mg^2+^ concentration are critical factors for incision on the Q-GR adduct, as observed previously for the nucleotide incision activity of APE1 (62,63,68,69). However, we were concerned that if the Q-GR adducts were unstable and were converted to AP sites under these acidic and low Mg^2+^ conditions, the products might be those produced by incision of Q-GR adduct-generated AP sites. We confirmed that the Q-GR adduct alone was stable under this reaction condition (0.1 mM MgCl_2_ at pH 6.8) and APE1 did not affect the stability of the Q-GR adduct in the presence of 10 mM EDTA and absence of 0.1 mM MgCl_2_ (Supplementary Figure S15D), demonstrating that the incision products were not those produced by the incision of Q-GR adduct-generated AP sites. Surprisingly, APE1 titration experiments at various incubation times revealed that the amount of enzyme required for incision reached saturation between 0.1 and 0.2 pmol (Figure 7D, E and Supplementary Figure S16), which represents a 1.6–3.3-fold molar excess of the enzyme over the total amount of dsDNA substrate (60 fmol) in the reaction. Further addition of the enzyme beyond 0.2 pmol did not further stimulate the reaction. Incision was dependent on only incubation time when saturating amounts of APE1 were added to the reactions, suggesting that a step after enzyme binding to DNA, such as a conformational change of the APE1-DNA complex or chemistry (72), occurred slowly, and that this step could be the rate limiting step in reactions containing saturating amounts of APE1. A graph plotted against the relative amounts of remaining T34Q-GR in reactions containing saturating amounts of APE1 (>0.2 pmol) versus the incubation times revealed a biphasic reaction rate (Figure 7F). The amount of T34Q-GR decreased with a half-life shorter than 9.2 min within the first 5 min and then decreased further but more slowly with a half-life of 17.3 min (Figure 7F). The biphasic reaction profiles of nucleotide incision reactions have been previously reported (73). The estimated reaction rates were >0.075 and 0.040 min^−1^ during the first phase and second phase, respectively. To confirm the incision site, the assay was performed using the 3′-labelled substrate mixture derived from T34Q-GR, T34Q and T34O (Figure 7G). The results demonstrated that APE1 generated two major products: a band migrating more slowly than the T26G-C marker and a band corresponding to the product generated from the 3′-labelled T34O (Figure 7G). These results strongly suggested that APE1 incises DNA 5′ to the Q-GR adduct, generating a 3′-OH and a P-Q-GR moiety at the 5′-end.

We also tested ExoIII under the incision assay condition (0.1 mM MgCl_2_ at pH 6.8). When the 5′-^32^P-labelled substrate was used, we could not evaluate whether the Q-GR adduct was incised because of the robust exonuclease activity (Supplementary Figure S17B). However, using the 3′-labelled substrates, we clearly detected the incision product of the Q-GR adduct as a band migrating more slowly than that of the T26G-C marker (Supplementary Figure S17C). Again, the incision activity was higher under the incision assay condition than under the AP endonuclease assay condition (10 mM MgCl_2_ at pH 7.5) (Supplementary Figure S17C and D).

**Figure 8.**
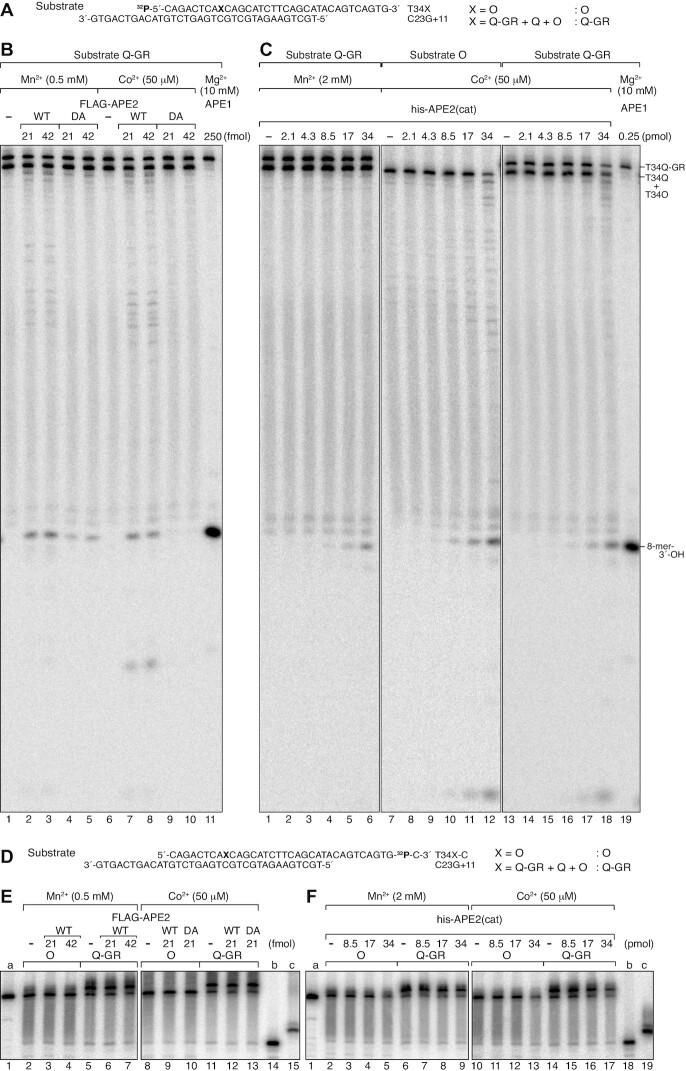
Nuclease assays of APE2. (**A**) Nucleotide sequences of the 5′-^32^P-labelled substrates used in (B) and (C). T34O and a mixture of T34Q-GR (50%), T34Q (10%), and T34O (40%) were annealed with C23G+11 to generate the substrates designated as ‘O’ and ‘Q-GR’, respectively. (**B**) The indicated substrates were incubated with the indicated amounts of FLAG-APE2 (WT) or FLAG-APE2(D227A) (DA) at 30°C for 1 hour in the presence of the indicated metal ions. Reaction products were resolved by 20% urea–PAGE. A control APE1 reaction under the 10 mM MgCl_2_ at pH 7.5 reaction condition was loaded as a size marker for the incision product 8-mer-3′-OH (lane 11). (**C**) The indicated substrates were incubated with the indicated amounts of his-APE2(cat) at 30°C for 1 hour in the presence of the indicated metal ions. The reaction products were resolved by 20% urea–PAGE. A control APE1 reaction under the 10 mM MgCl_2_ at pH 7.5 reaction condition was loaded as a size marker for the incision product 8-mer-3′-OH (lane 19). (**D**) Nucleotide sequences of the 3′-^32^P-labelled substrates used in E-F. T34O and a mixture of T34Q-GR (76%), T34Q (17%), and T34O (7%) were 3′-labelled with [α-^32^P]dCTP to generate the substrates designated as ‘O’ and ‘Q-GR’, respectively. (**E, F**) Nuclease assays of APE2 with 3′-^32^P-labelled substrates. The indicated substrates were incubated with FLAG-APE2 (WT) or FLAG-APE2(D227A) (DA) (E) or his-APE2(cat) (F) at 30°C for 1 hour in the presence of the indicated metal ions. The reaction products were resolved by 20% urea–PAGE. The size markers, a, b and c are shown in Figure 6G.

We next examined the activity of a second human AP endonuclease, APE2 (74,75). Here, we used dsDNA substrates containing 11-base 3′-overhangs to prohibit the robust exonuclease activity of APE2 (Figure 8A). We first tested N-terminally FLAG-tagged full-length APE2 (FLAG-APE2) overexpressed in and partially purified from HEK293 cells (Supplementary Figure S18B). We found that FLAG-APE2 generated an 8-mer-3′-OH product in a reaction containing Mn^2+^ at pH 7,5 (Figure 8B, lanes 2–3), a condition under which the nuclease activity of APE2 has been previously clearly detected (36,76), corresponding to the product produced by APE1 (Figure 8B, lane 11). However, we suspect that contaminating AP endonucleases, possibly including APE1, were present because a small amount of the 8-mer-3′-OH product was detected in reactions with the nuclease deficient mutant, FLAG-APE2(D277A) (36) (Figure 8B, lanes 4–5). We found that the 8-mer-3′-OH product was also generated in reactions containing a low concentration of Co^2+^ (Figure 8B, lanes 7–8). Interestingly, Co^2+^ specifically stimulated the activity of FLAG-APE2, but not that of the contaminating AP endonucleases (Figure 8B, lanes 9–10). To confirm these results, and exclude the effects of the contaminating AP endonucleases, we purified a recombinant catalytic domain of APE2 (amino acids 1–361) with a histidine tag at its N-terminus (his-APE2(cat)) from the ExoIII/EndoIV-deficient *E. coli* strain RPC501 (Supplementary Figure S18A). Purified his-APE2(cat) exhibited Co^2+^-dependent nuclease activity on a substrate containing T34O (Figure 8C, lanes 7–12) and Mn^2+^- and Co^2+^-dependent nuclease activity on a substrate mixture containing T34Q-GR, T34Q and T34O (Figure 8C, lanes 1–6 and 13–18), although the activity was reduced approximately 1000-fold compared with that of full-length FLAG-APE2, possibly due to the absence of the C-terminal Zf-GRF domain (76). We confirmed that the Co^2+^-dependent nuclease activity was intrinsic to his-APE2(cat) protein and that contaminating nucleases were absent from the preparation, because the activity of his-APE2(cat) coincided exactly with the elution profile of his-APE2(cat) protein from the gel filtration column used in the final step of purification (Supplementary Figure S18C). Although his-APE2(cat) clearly generated an 8-mer-3′-OH product in the presence of Co^2+^ (Figure 8C, lanes 7–18), we could not evaluate whether the Q-GR adduct or AP site was incised by the endonuclease activity of his-APE2(cat) because of robust exonuclease-mediated degradation (Figure 8C, lanes 7–18). To obtain direct evidence of cleavage 5′ to the Q-GR adduct, we used a 3′-labelled oligonucleotide (Figure 8D); however, no cleavage of 3′-labelled T34Q-GR in a mixture of substrates was detected with FLAG-APE2 (Figure 8E, lanes 5–7 and lanes 11–13) or his-APE2(cat) (Figure 8F, lanes 6–9 and 14–17), although the amount of the full-length substrate decreased (Figure 8F, lanes 14–17) and the amount of ^32^P-labeled dCMP excised from the 3′-end of the substrate concomitantly increased (data not shown) in an enzyme concentration-dependent manner, consistent with the results we obtained with the 5′-^32^P-labelled substrate (Figure 8C, lanes 13–18). Surprisingly, we also failed to detect the product of AP site cleavage (Figure 8E, lanes 2–4 and 8–10, and 8F, lanes 2–5 and 10–13). These results suggested that the 5′-^32^P labelled 8-mer-3′-OH product detected in reactions with APE2 (Figure 8B and C) is not a true cleavage product of the lesions but is produced during pausing of the exonuclease reactions. Collectively, we conclude that APE2 has poor endonuclease activity on the Q-GR adduct.

Lastly, we examined TDP1 because its phosphodiesterase activity cleaves the AP site in dsDNA and ssDNA to leave a 3′-phosphate, although it preferentially cleaves ssDNA (77–79). A histidine tagged recombinant TDP1 (his-TDP1) was overproduced in and purified from RPC501 (Supplementary Figure S19A). We confirmed that his-TDP1 incised the AP site in dsDNA, as reported previously in the absence of divalent cations at pH 7.5 (77–79) (Supplementary Figure S19), but did not incise the Q-GR adduct in dsDNA or ssDNA (Supplementary Figure S19). This finding suggested that the conversion of AP sites to thiazolidine-adducts in ssDNA prevents cleavage by TDP1.

## DISCUSSION

DDT pathways that act in the presence of an AP site are crucial for cell maintenance. Recently, it was discovered that HMCES reacts with the aldehyde form of AP sites in ssDNA at replication forks, generating a stable thiazolidine protein-DNA crosslink (20,22–24). This stable HMCES-crosslink prevents spontaneous and nuclease-dependent breakage of the replication fork, and is subsequently resolved by proteasome-mediated degradation (20). However, it remains to be determined how HMCES-crosslinked ssDNA and/or proteasome-degraded HMCES adducts are processed to restore intact dsDNA. In the present study, we explored these processes using model substrates of HMCES-crosslinked DNA and protease-digested HMCES adducts *in vitro*.

### Quantitative analysis of peptide-conjugated 4-carboxythiazolidine adducts

A model of proteasome-degraded HMCES-crosslinked DNA adducts was created by digesting HMCES-crosslinked oligonucleotides with proteinase K. The reaction products were a mixture of 4-carboxythiazolidine-containing oligonucleotides, in which peptide fragments of various lengths were conjugated to the 4-carboxythiazolidine moiety because of incomplete digestion by proteinase K. To determine the identity of these peptides, we developed a novel method for the quantification of cysteine and cysteine-containing peptides. First, peptides containing cysteine were cleaved from the oligonucleotide by heating. Second, the peptides were reacted with NBM so that they could be retained on a reversed-phase HPLC column and detected by UV absorption. The addition of TCEP·HCl, a reducing agent that cleaves the disulfide bond, prevented the reaction of peptides with dissolved oxygen, which could affect quantitative analyses. Since the Michael addition of a thiol to maleimide produces racemates, this reaction produced two enantiomers, which were separated on a reversed-phase column. Subsequent LC–MS experiments revealed a pair of peaks exhibiting the same *m*/*z* values. This is a good indicator that we could identify NBM-reacted peptides, in the order of pmol amounts, from background noise. This procedure allowed us to determine the amounts of 4-carboxythiazolidine and peptide-conjugated 4-carboxythiazolidine adducts.

### Stability of HMCES-crosslink and 4-carboxythiazolidine adducts

The 4-carboxythiazolidine adduct itself and the 4-carboxythiazolidine-conjugated Gly-Arg peptide (Q-GR) are relatively resistant to alkaline treatment; however, they are heat labile and spontaneously convert to an AP site and cysteine or the Cys-Gly-Arg peptide. The half-life of Q-GR was 20–24 (in ssDNA) or 12 (in dsDNA) hours at 37°C and pH 7.5, indicating that the reaction is reversible under normal conditions. Recently, it was reported that HMCES-crosslinks in ssDNA are also reversible (24). Interestingly, we found that HMCES-crosslinks are destabilised in dsDNA, with a half-life of approximately 3.5 hours at 30°C. This half-life is much shorter than that of the Q-GR adduct, suggesting that the de-crosslinking reaction is enzymatic. In the ssDNA-HMCES crosslink complex, the ssDNA backbone is constrained, resulting in a kink at the AP site produced by interactions between adjacent nucleobases and amino acid residues of HMCES (22,23). The molecular mechanism of the interaction suggests that the presence of a complementary strand prevents such a ssDNA-HMCES interaction and thus dsDNA formation and HMCES binding are mutually exclusive (22,23). We consider the crosslinking and de-crosslinking reactions in the ssDNA-HMCES complex to be in equilibrium, although the equilibrium is largely shifted in favour of the crosslink reaction in crystals (22,23). After de-crosslinking, the de-crosslinked products, HMCES and AP site-containing ssDNA, may still be present as non-covalent complexes because of the high affinity of the interaction between ssDNA and HMCES (20,22,23). Thus, the re-crosslinking reaction could predominate over the dissociation of HMCES from ssDNA, although a small fraction is indeed dissociated as reported recently (24). By contrast, in the presence of a complementary strand, de-crosslinked ssDNA could readily form dsDNA in the vicinity of the AP site and eventually induce dissociation of HMCES due to abrogation of the interactions between nucleobases and amino acid residues of HMCES, thereby preventing re-crosslinking. It is possible that the dissociation of crosslinked HMCES via the reverse reaction after the formation of dsDNA at the site of the adduct through the activity of the DDT allows the repair of the regenerated AP site by BER.

### TLS of HMCES-crosslinks and 4-carboxythiazolidine adducts

Replication-blocking lesions induce ssDNA regions at replication forks. In the leading strand, long ssDNA regions arise when pol ϵ encounters a lesion because DNA helicase activity becomes uncoupled from DNA synthesis. In the lagging strand, ssDNA regions arise as gaps between Okazaki fragments (80). In these cases, HMCES-crosslinks would be generated next to the stalled primer termini. It is also possible that HMCES interacts with AP sites in ssDNA generated during a normal cycle of Okazaki fragment synthesis before pol δ encounters the AP site. Here, HMCES-crosslinks would be generated between two Okazaki fragments, which is far in front of the primer end of ongoing Okazaki fragment synthesis. In this study we examined both situations.

We have demonstrated that replicative and TLS polymerases that encounter HMCES-crosslinks stall at several bases upstream of the crosslinked site, which might reflect what happens on the HMCES-crosslinked lagging strand during a normal cycle of Okazaki fragment synthesis. We also demonstrated that the primer end located next to the crosslinking site was rarely extended beyond the HMCES-crosslink, which might reflect what happens after replicative polymerases encounter AP sites. The Q-GR adduct, which could mimic a proteasome-degraded HMCES lesion, blocked replication to a similar extent as the AP site. Pol δ, pol η and pol κ predominantly inserted A opposite the Q-GR adduct, as observed at AP sites. Importantly, unlike the AP site, the Q-GR adduct was not a good substrate for the dCMP transferase activity of REV1, suggesting that the conversion of AP sites to 4-carboxythiazolidine adducts abrogates ‘C-rule insertion’ of AP site-induced mutations.

### Repair of 4-carboxythiazolidine adducts

We predicted that 4-carboxythiazolidine adducts would be repaired by BER, which could be initiated by introducing an incision 5′ to the lesion by AP endonucleases. *E. coli* encodes two AP endonucleases, ExoIII and EndoIV (57). The human genome also encodes two AP endonucleases, APE1 and APE2, which belong to the ExoIII family of proteins (74). APE1 and ExoIII are two closely related endonucleases, whereas APE2 is a member of a separate subfamily (74). EndoIV belongs to another family distinct from the Exo III family, and this EndoIV family of proteins is absent in humans (74). We found that human APE1 and *E. coli* ExoIII and EndoIV can incise DNA 5′ to the Q-GR adduct. Interestingly, APE1 and ExoIII, two closely related enzymes, are active under nucleotide incision reaction condition of low pH and low Mg^2+^ (62,63,68–71). The reaction rates of APE1 for different damage bases under the nucleotide incision reaction condition are reported to range from 0.1 to 1.0 min^−1^ in most cases (63,72,81) (summarised in ref 82), which are close to the reaction rate of >0.04 min^−1^ for the Q-GR adduct. Since nucleotide incision reactions are physiologically relevant (81,83), thiazolidine-adducts could be incised by APE1 *in vivo*, considering that the cellular concentration of APE1 is 0.1∼1 μM, based on an estimate that each human fibroblast contains approximately 3 × 10^5^ molecules of APE1 (84,85). After the incision, the incision could be repaired by long-patch BER because the excision of the Q-GR moiety is resistant to β-elimination (86,87). Alternatively, the spontaneous conversion of 4-carboxythiazolidine adducts to AP sites, which is stimulated in dsDNA, could lead to repair by mostly short-patch BER (87,88).

### Hypothetical repair pathways for HMCES-crosslinked DNA

**Figure 9.**
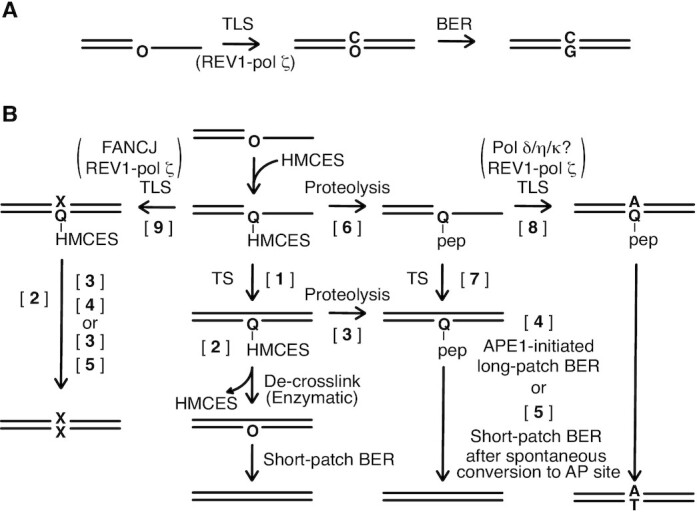
DNA damage tolerance pathways of AP site. (**A**) REV1 and pol ζ-dependent TLS through AP sites. The catalytic activity of REV1 is required for the insertion of dCMP opposite the lesion. (**B**) HMCES-dependent hypothetical DDT and subsequent repair pathways for AP sites. [**1**] HMCES-crosslink induces TS. [**2**] HMCES catalyses the reversal of its own crosslinking in dsDNA. The resultant AP site is repaired by mostly short-patch BER. [**3**] HMCES-crosslink is degraded by the proteasome after TS is completed. It is possible that proteasome degradation leaves a short peptide fragment attached to the 4-carboxythiazolidine adduct, indicated as Q-pep. [**4**] The resultant Q-pep adduct is repaired by APE1-initiated long-patch BER. [**5**] The spontaneous reversal of Q-pep adduct generates an AP site and releases the peptide. The resultant AP site is repaired by mostly short-patch BER. [**6**] HMCES crosslinked to ssDNA is degraded by the proteasome. Replication through the resultant Q-pep adduct is achieved by TS [**7**] or TLS [**8**]. Since the extent to which each polymerase contributes to TLS is unknown, dAMP insertion opposite the lesion is shown. It is likely that TLS through Q-pep adduct is dependent on pol ζ and the non-catalytic activity of REV1. [**9**] FANCJ-dependent TLS. The nucleotide incorporated opposite the HMCES-crosslink is unknown. After conversion to dsDNA by TLS, the HMCES-crosslinked dsDNA is repaired by a pathway similar to that in [**2**], [**3**], and [**4**] or [**3**] and [**5**].

Figure 9A shows the canonical TLS pathway operating at AP sites, which is dependent on REV1 and pol ζ. The dCMP transferase activity of REV1 is required for ‘C-rule insertion’ (10,13–19,55,89). Figure 9B illustrates the hypothetical HMCES-dependent repair pathways functioning at AP sites. Because we have shown that the HMCES-crosslink strongly blocks DNA synthesis, it is possible that it acts as a signal for TS, an error-free sub-pathway of DDT (Figure 9B, pathway [1]). This is consistent with previous reports that HMCES prevents error-prone TLS and protects cells from AP site toxicity (20,21,25,26). Interestingly, a major fraction of AP sites is bypassed by TS in yeast (12). After TS is completed, HMCES can reverse the crosslinking through its intrinsic enzyme activity. The resulting AP site can be subsequently repaired by mostly short-patch BER (87,88) (Figure 9B pathway [2]). HMCES crosslinked to dsDNA could be degraded by the proteasome (Figure 9B pathway [3]). Since it has not been determined whether proteasome degradation leaves a small peptide fragment i.e. generates 4-carboxythiazolidine-peptide conjugate adducts, the proteasome-degradation products are designated tentatively as Q-pep (Figure 9B). The resultant Q-pep adducts are repaired by the APE1-initiated long-patch BER because the adducts are resistant to β-elimination (86,87) (Figure 9B, pathway [4]). Alternatively, following spontaneous conversion of the 4-carboxythiazolidine adducts, the resulting AP site is repaired by mostly short-patch BER (87,88) (Figure 9B, pathway [5]). Once HMCES crosslinked to ssDNA is degraded by the proteasome (Figure 9B, pathway [6]), it is possible that the resulting 4-carboxythiazolidine-peptide adduct-containing DNA is replicated by TS (Figure 9B, pathway [7]) or by TLS, which could be dependent on pol ζ and REV1 (Figure 9B, pathway [8]). The efficiency of TLS across 4-carboxythiazolidine adducts would be equivalent to that across AP sites. While the catalytic activity of REV1 involved in the insertion of dCMP opposite 4-carboxythiazolidine adducts is weaker than that at AP sites, due to its inefficiency, the non-catalytic function of REV1 might still be required (34,89,90). Because spontaneous depurination of guanine is a major source of AP sites, inefficient dCMP incorporation opposite 4-carboxythiazolidine adducts could be more mutagenic than AP sites. Therefore, TLS across 4-carboxythiazolidine adducts may be only a minor pathway.

While this manuscript was under revision, it was reported that FANCJ helicase can unfold the HMCES crosslink to promote TLS by REV1-pol ζ (91); thus, we have added it to our model (Figure 9B, pathway [9]). However, the contribution of the FANCJ pathway may be small, because error-free TS is considered to be the major DDT pathway for bypass replication across HMCES-crosslinks in human cells (20,21,26,34). More research will be required to determine whether the HMCES crosslink induces TS, and, if so, which signal facilitates the switch to TS. Further work will also be required to determine how the HMCES-dependent DDT pathway integrates into the regulatory network of PCNA ubiquitination (92–95), and whether the proteasomal degradation of HMCES is initiated in ssDNA or after its conversion to dsDNA. The identity of a potential E3 ligase involved in the ubiquitination of crosslinked HMCES will also need to be investigated (90). It also remains to be determined how human SPRTN contributes to the degradation of crosslinked HMCES. Together, this research will lead to a better understanding of the physiological roles of the HMCES-dependent DDT pathway operating at AP sites.

## DATA AVAILABILITY

The data underlying this article will be shared on reasonable request to the corresponding author.

## Supplementary Material

gkad246_Supplemental_Files

## Notes

Present address: Yohei Sugimoto, Division of Molecular Oncology, Center for Neurological Diseases and Cancer, Nagoya University Graduate School of Medicine, 65 Tsurumai-cho, Showa-ku, Nagoya 466-8550, Japan.
