# Supplementary material for: Novel mechanisms for the removal of strong replication-blocking HMCES- and thiazolidine-DNA adducts in humans
Source: Nucleic Acids Res. 2023 Apr 6;51(10):4959–81. doi: 10.1093/nar/gkad246 (PMC10250235; doi:10.1093/nar/gkad246)
Supplement: gkad246_Supplemental_Files [file gkad246_supplemental_files.zip › Revised Supplementary Figures.pdf]

Novel mechanisms for the removal of strong replication-blocking HMCES- and thiazolidine-DNA adducts in humans

Yohei Sugimoto[1,2,†], Yuji Masuda[1,2,*,†], Shigenori Iwai[3], Yumi Miyake[4], Rie Kanao[1,2], and Chikahide Masutani[1,2]

[1]Department of Genome Dynamics, Research Institute of Environmental Medicine, Nagoya University, Furo-cho, Chikusa-ku, Nagoya 464-8601, Japan

[2]Department of Molecular Pharmaco-Biology, Nagoya University Graduate School of Medicine, 65 Tsurumai-cho, Showa-ku, Nagoya 466-8550, Japan

[3]Graduate School of Engineering Science, Osaka University, 1-3 Machikaneyama, Toyonaka, Osaka, 560-8531, Japan

[4]Forefront Research Center, Graduate School of Science, Osaka University, 1-1 Machikaneyama, Toyonaka, Osaka 560-0043, Japan

*To whom correspondence should be addressed. Tel: 81-52-789-3871; Fax: 81-52-789-3890; Email: masuda@riem.nagoya-u.ac.jp

†The authors wish it to be known that, in their opinion, the first two authors should be regarded as Joint First Authors.

Present Address: Yohei Sugimoto, Division of Molecular Oncology, Center for Neurological Diseases and Cancer, Nagoya University Graduate School of Medicine, 65 Tsurumai-cho, Showa-ku, Nagoya 466-8550, Japan

Supplementary Figures S1-S19

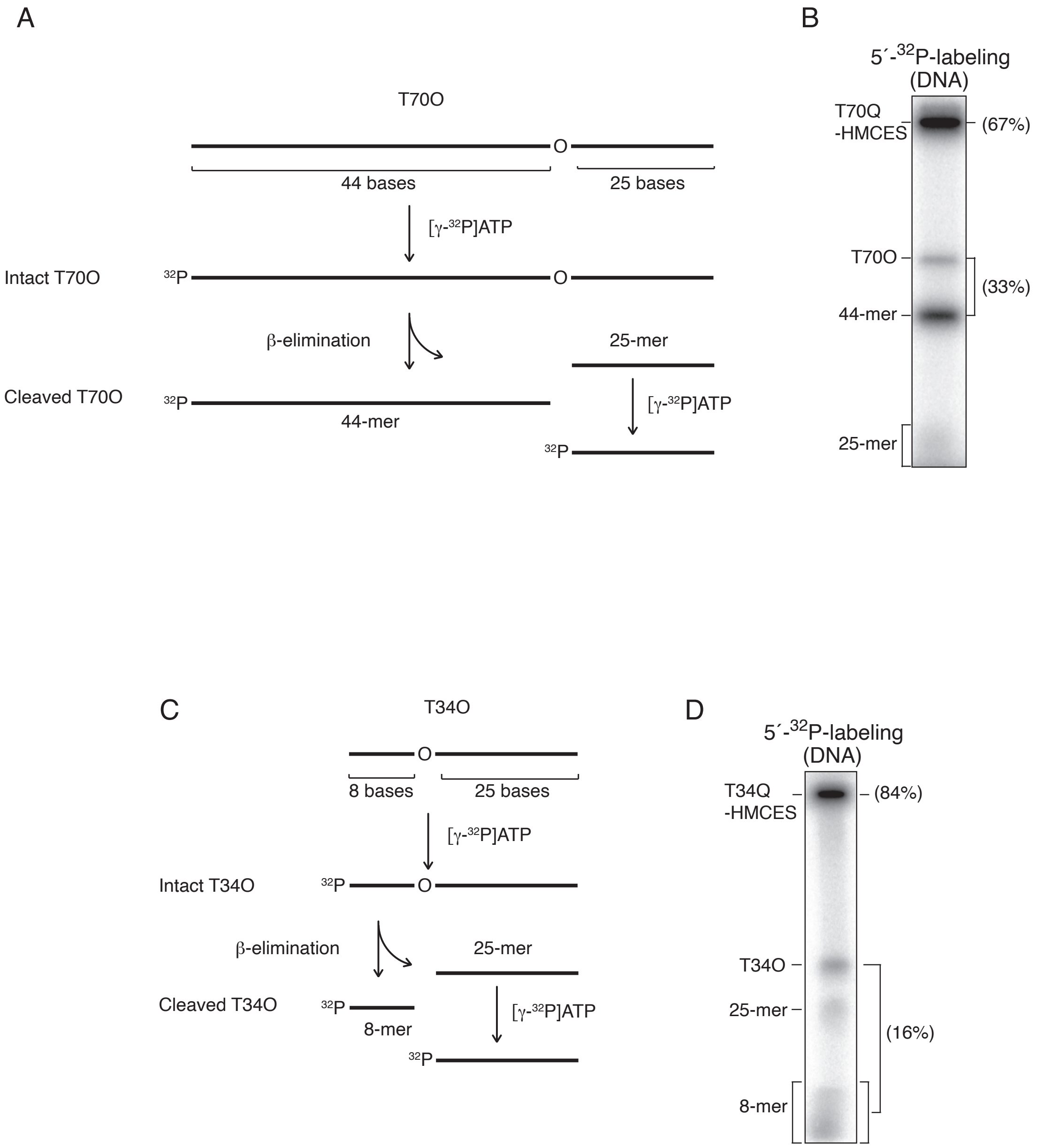

Supplementary Figure S1. Estimation of the relative amounts of contaminating T70O and T34O oligonucleotides in the purified T70Q-HMCES and T34Q-HMCES fractions, respectively.

A. Schematic of the labelling reaction of the contaminating T70O oligonucleotide with T4 polynucleotide kinase and [γ-^{32}P]ATP in purified T70Q-HMCES fractions. A significant fraction of T70O was cleaved by β-elimination of the AP site. Since T70O was stable by SDS-PAGE analysis (Figure 1B), cleavage occurred during this kinase reaction. How β-elimination of the AP site was facilitated in this kinase reaction has not been addressed, because we were able to estimate the relative amount of contaminating T70O despite the partial cleavage.

B. 20% SDA-PAGE analysis of the labelling sample. The total fraction of the contaminating T70O was estimated as the sum of T70O and the cleaved 44-mer oligonucleotide.

C. Schematic of the labelling reaction of the contaminating T34O oligonucleotide in the purified T34Q-HMCES fraction with T4 polynucleotide kinase and [γ-^{32}P]ATP.

D. 30% SDA-PAGE analysis of the labelled sample. The total fraction of the contaminating T34O was estimated as the sum of T34O and the cleaved 8-mer oligonucleotide.

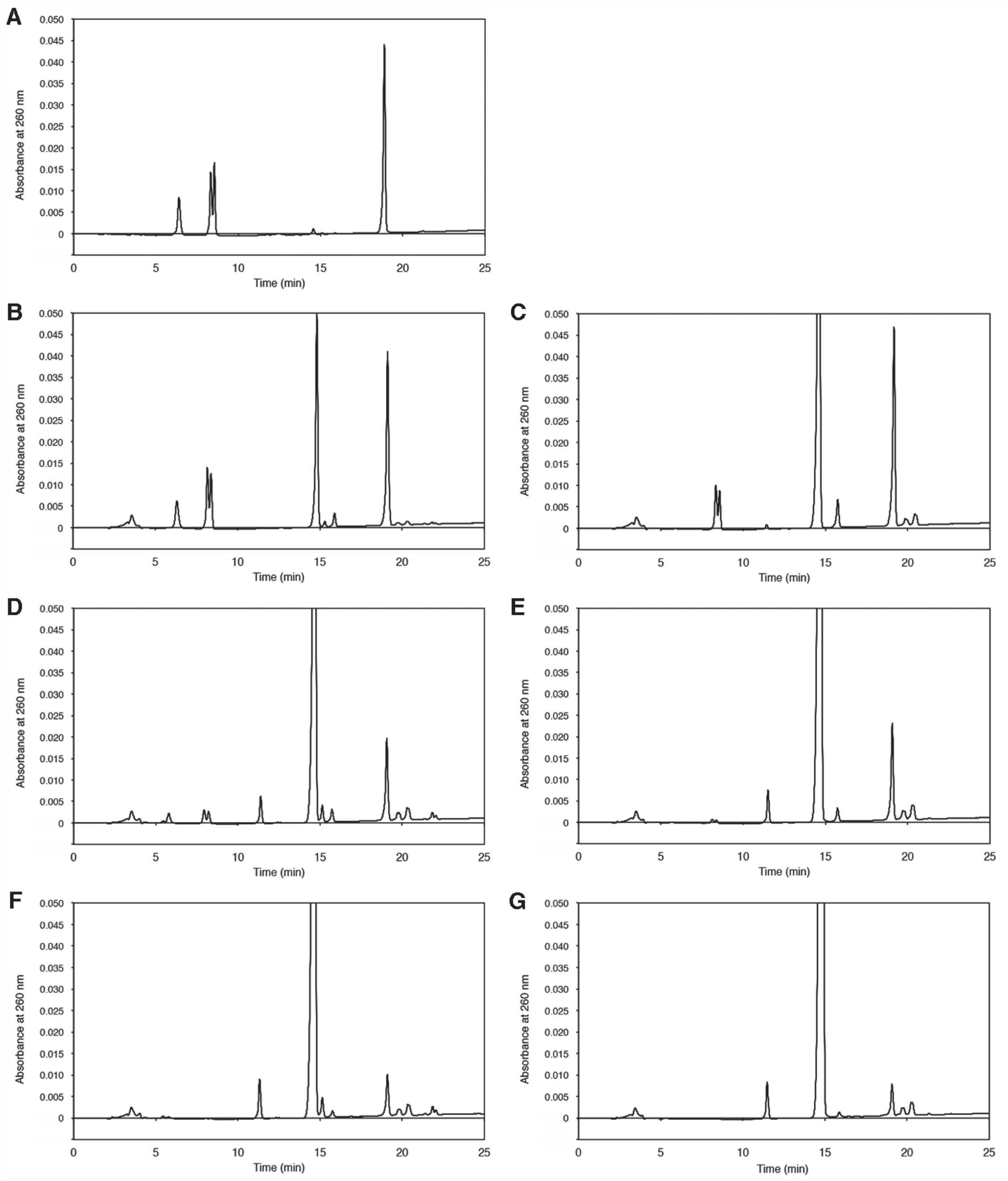

Supplementary Figure S2. Analysis of the reaction of cysteine with NBM. Cysteine (100 nmol) was treated with NBM (500 nmol) in the presence (**A**, **B**, **D**, and **F**) or absence (**C**, **E**, and **G**) of TCEP·HCl (100 nmol) in water (**A**) or 50 mM sodium phosphate (pH 6.0, **B** and **C**; pH 7.0, **D** and **E**; pH 7.5, **F** and **G**) containing 10% ethanol (50 μL) at 25°C for 24 h. Aliquots (5 μL) were analysed using an Inertsil ODS-3 column at a flow rate of 1.0 mL/min with a linear gradient of 19–76% acetonitrile containing 0.1% formic acid for 20 min. The peaks with retention times of 6.4 min, 8.3 and 8.5 min, and 18.9 min correspond to compounds **4**, **3**, and **2** in Figure 1E, respectively.

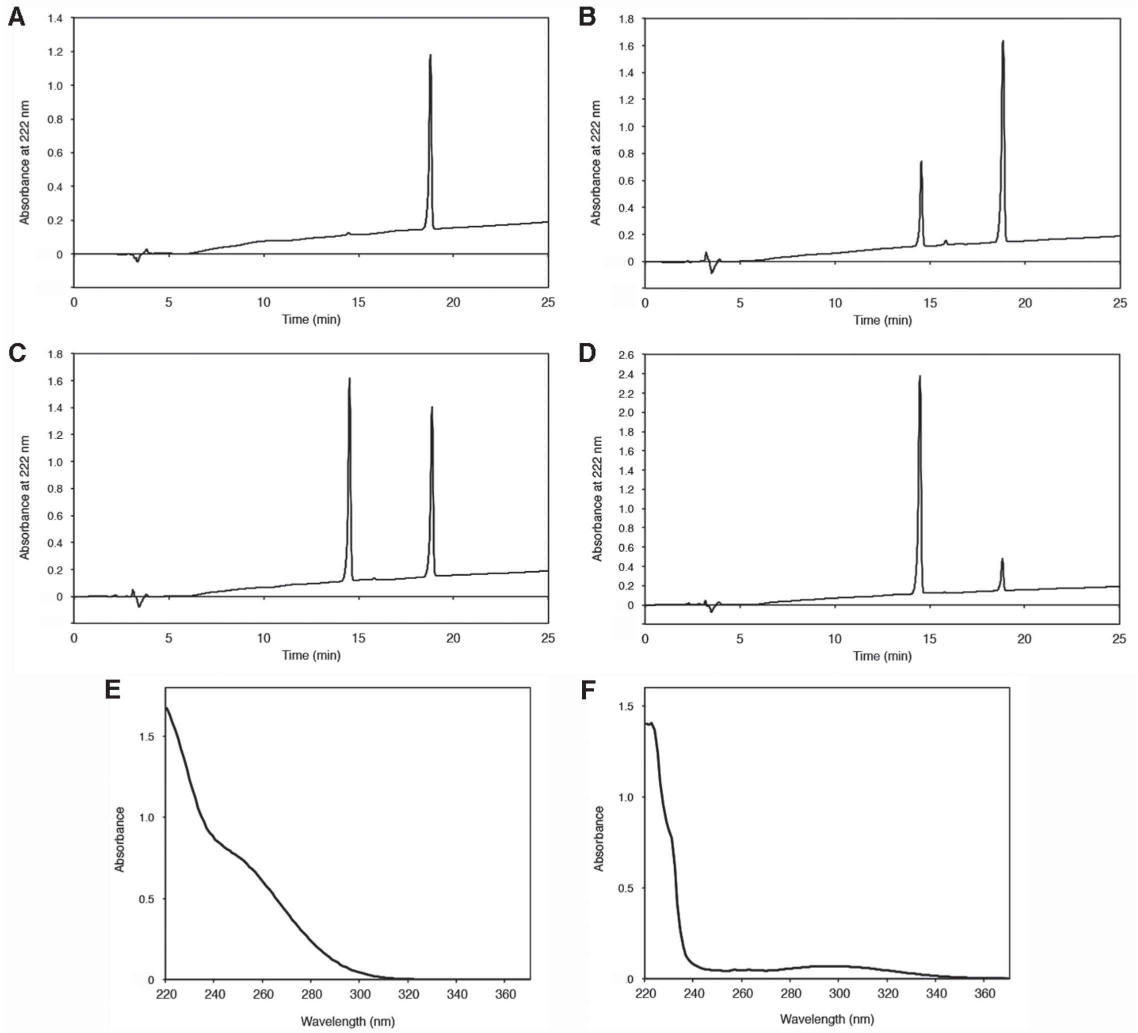

Supplementary Figure S3. Influence of sodium phosphate on NBM. (**A**–**D**) Conversion of NBM to an unknown product. A 100 mM solution of NBM in ethanol (5 μL) was mixed with water (**A**) or 50 mM sodium phosphate (pH 6.0, **B**; pH 7.0, **C**; pH 7.5, **D**) (45 μL), and the mixtures were kept at 25°C for 24 h. HPLC analysis was performed in the same manner as described in the legend to Supplementary Figure S2. Since the molar extinction coefficients are largely different between the two compounds, chromatograms monitored at a short wavelength are shown. (**E** and **F**) UV absorption spectra of the compounds detected at 14.5 min (**E**) and 18.9 min (**F**) in panel **C**. The latter is NBM.

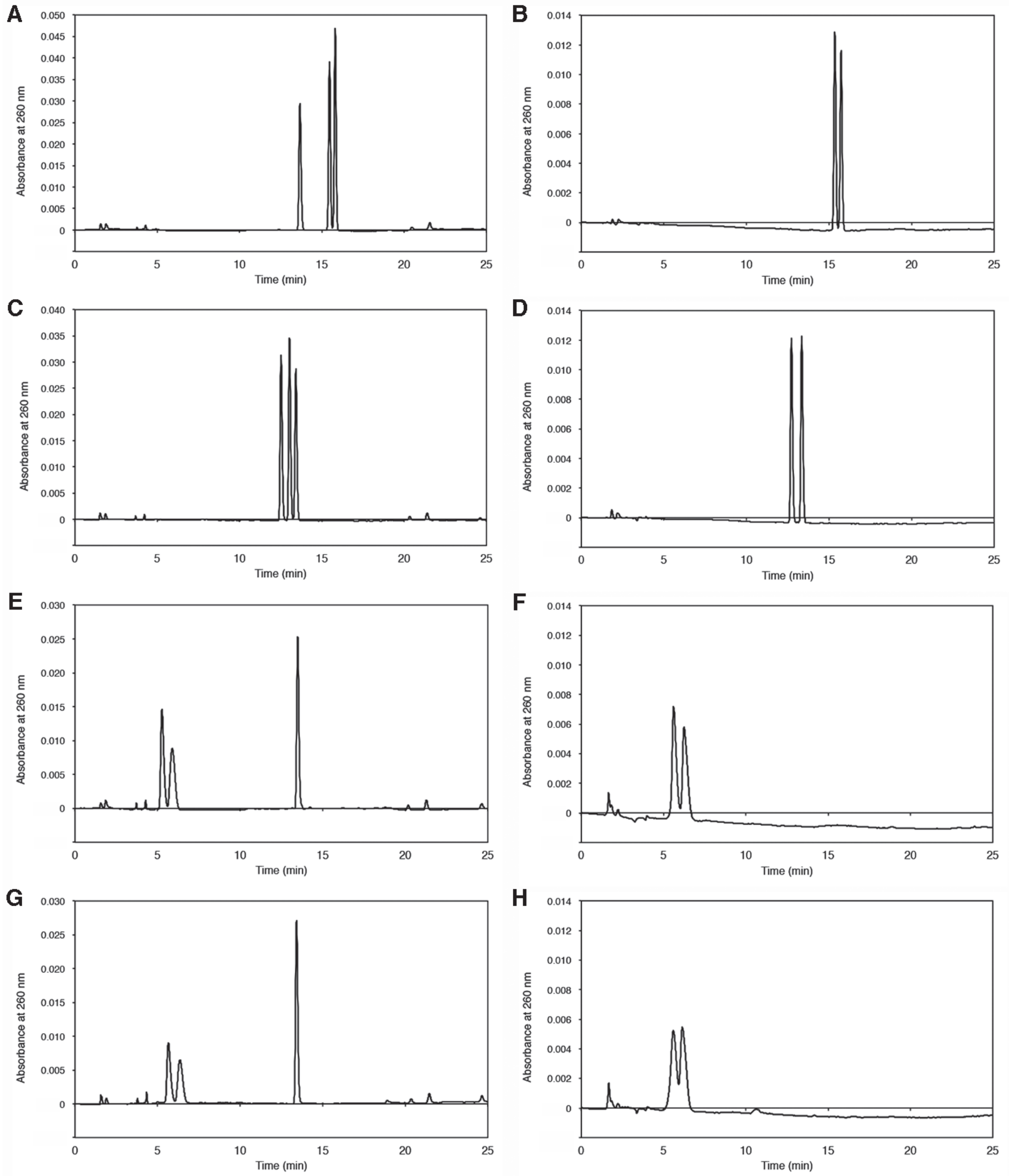

Supplementary Figure S4. Preparation of the reference materials. Cysteine (**A** and **B**), CG (**C** and **D**), CGR (**E** and **F**), and CGRT (**G** and **H**) were treated with NBM in water in the presence of TCEP·HCl, as described in MATERIALS AND METHODS. The reaction mixtures (**A**, **C**, **E**, and **G**) were analysed using an Inertsil ODS-3 column at a flow rate of 1.0 mL/min with a linear gradient of 9.5–38% acetonitrile containing 0.1% formic acid for 20 min. In addition to the desired products, the peak of compound **4** (Figure 1E) was detected at 13.5 min. After purification under the same HPLC conditions, the purified compounds were analysed again (**B**, **D**, **F**, and **H**).

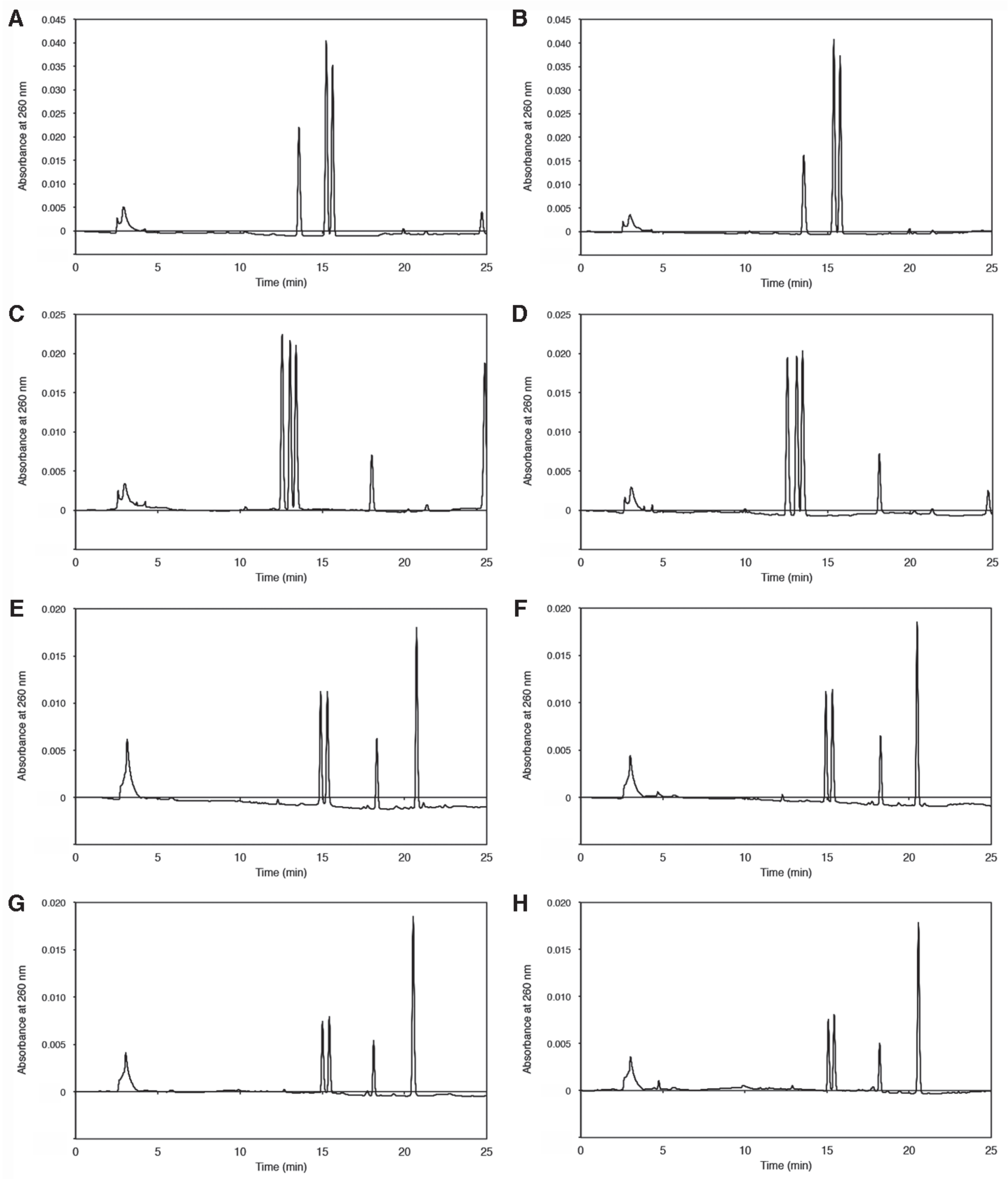

Supplementary Figure S5. Analysis of the thermostability of the peptides. Cysteine (**A** and **B**), CG (**C** and **D**), CGR (**E** and **F**), and CGRT (**G** and **H**) (100 nmol) were dissolved in 10 mM HEPES-NaOH (pH 7.5, 25 μL). One of the two solutions of each compound was kept at room temperature (**A**, **C**, **E**, and **G**), and the other was heated at 95°C for 10 min (**B**, **D**, **F**, and **H**). After drying, the residues were treated with TCEP (100 nmol) and NBM (250 nmol) in 100 mM sodium phosphate (pH 6.0). The reaction mixtures were analysed by HPLC using an Inertsil ODS-3 column at a flow rate of 1.0 mL/min with a linear gradient of 9.5–38% (**A**–**D**) or 0–28.5% (**E**–**H**) acetonitrile containing 0.1% formic acid for 20 min.

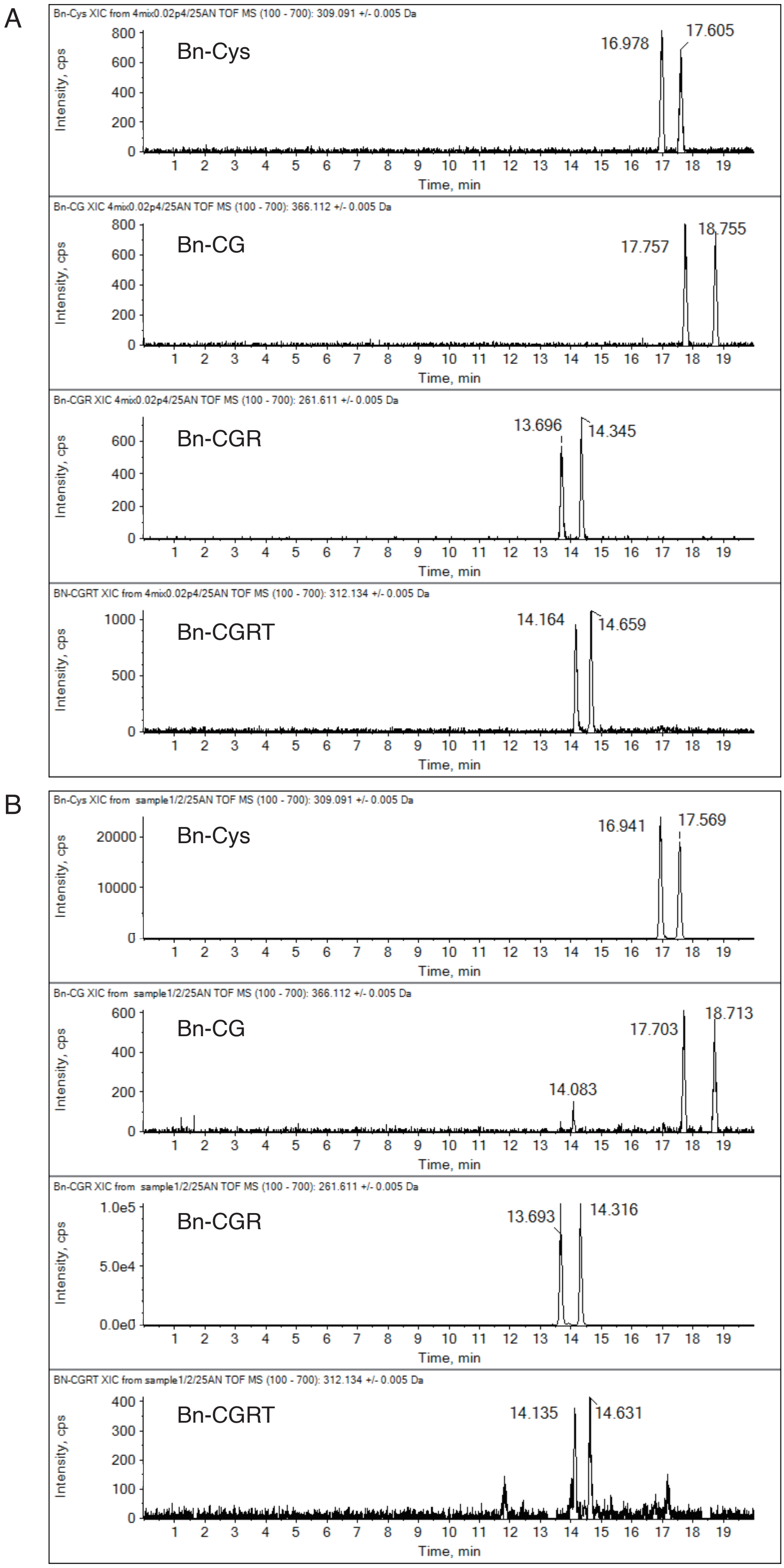
A
Bn-Cys XIC from 4mix0.02p4/25AN TOF MS (100 - 700): 309.091 +/- 0.005 Da
Bn-Cys
16.978
17.605
Bn-CG XIC 4mix0.02p4/25AN TOF MS (100 - 700): 366.112 +/- 0.005 Da
Bn-CG
17.757
18.755
Bn-CGR XIC 4mix0.02p4/25AN TOF MS (100 - 700): 261.611 +/- 0.005 Da
Bn-CGR
13.696
14.345
BN-CGRT XIC from 4mix0.02p4/25AN TOF MS (100 - 700): 312.134 +/- 0.005 Da
Bn-CGRT
14.164
14.659
B
Bn-Cys XIC from sample1/2/25AN TOF MS (100 - 700): 309.091 +/- 0.005 Da
Bn-Cys
16.941
17.569
Bn-CG XIC from sample1/2/25AN TOF MS (100 - 700): 366.112 +/- 0.005 Da
Bn-CG
17.703
18.713
14.083
Bn-CGR XIC from sample1/2/25AN TOF MS (100 - 700): 261.611 +/- 0.005 Da
Bn-CGR
13.693
14.316
BN-CGRT XIC from sample1/2/25AN TOF MS (100 - 700): 312.134 +/- 0.005 Da
Bn-CGRT
14.135
14.631
Intensity, cps
Time, min

Supplementary Figure S6. The extracted ion chromatograms. Each extracted ion chromatogram was created with 0.01 Da mass window for Bn-Cys (*m/z* 309.091), Bn-CG (*m/z* 366.112), Bn-CGR (*m/z* 261.611), and Bn-CGRT (*m/z* 312.134).

A. The standard reference materials were dissolved in acetonitrile/water (25:75) at a concentration of 0.02 μmol/L.

B. The T34Q-X sample dissolved in acetonitrile/water (25:75). The derivatised T34Q-X sample was dissolved in 100 μL of acetonitrile/water (50:50) and then diluted with the same volume of water.

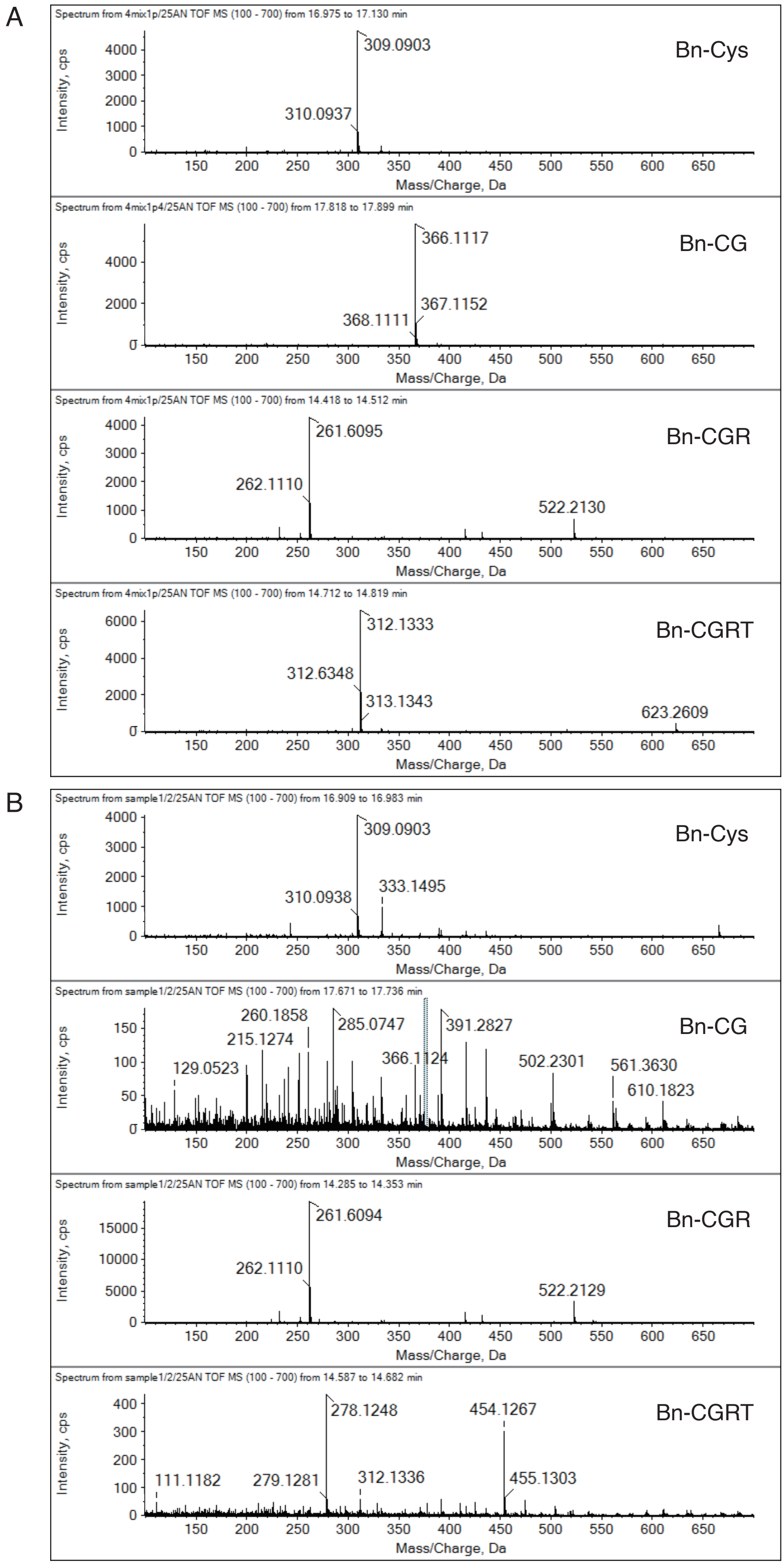
A
Spectrum from 4mix1p/25AN TOF MS (100 - 700) from 16.975 to 17.130 min
Bn-Cys
309.0903
310.0937
Spectrum from 4mix1p4/25AN TOF MS (100 - 700) from 17.818 to 17.899 min
Bn-CG
366.1117
367.1152
368.1111
Spectrum from 4mix1p/25AN TOF MS (100 - 700) from 14.418 to 14.512 min
Bn-CGR
261.6095
262.1110
522.2130
Spectrum from 4mix1p/25AN TOF MS (100 - 700) from 14.712 to 14.819 min
Bn-CGRT
312.1333
312.6348
313.1343
623.2609
B
Spectrum from sample1/2/25AN TOF MS (100 - 700) from 16.909 to 16.983 min
Bn-Cys
309.0903
310.0938
333.1495
Spectrum from sample1/2/25AN TOF MS (100 - 700) from 17.671 to 17.736 min
Bn-CG
260.1858
215.1274
285.0747
391.2827
129.0523
366.1124
502.2301
561.3630
610.1823
Spectrum from sample1/2/25AN TOF MS (100 - 700) from 14.285 to 14.353 min
Bn-CGR
261.6094
262.1110
522.2129
Spectrum from sample1/2/25AN TOF MS (100 - 700) from 14.587 to 14.682 min
Bn-CGRT
278.1248
454.1267
111.1182
279.1281
312.1336
455.1303
Intensity, cps
Mass/Charge, Da

Supplementary Figure S7. Mass spectra of NBM-derivatised peptides obtained from the peaks detected in the extracted ion chromatograms.

A. The standard mixed solution (1 μmol/L).

B. The T34Q-X sample dissolved in acetonitrile/water (25:75). The derivatised T34Q-X sample was dissolved in 100 μL of acetonitrile/water (50:50) and then diluted with the same volume of water.

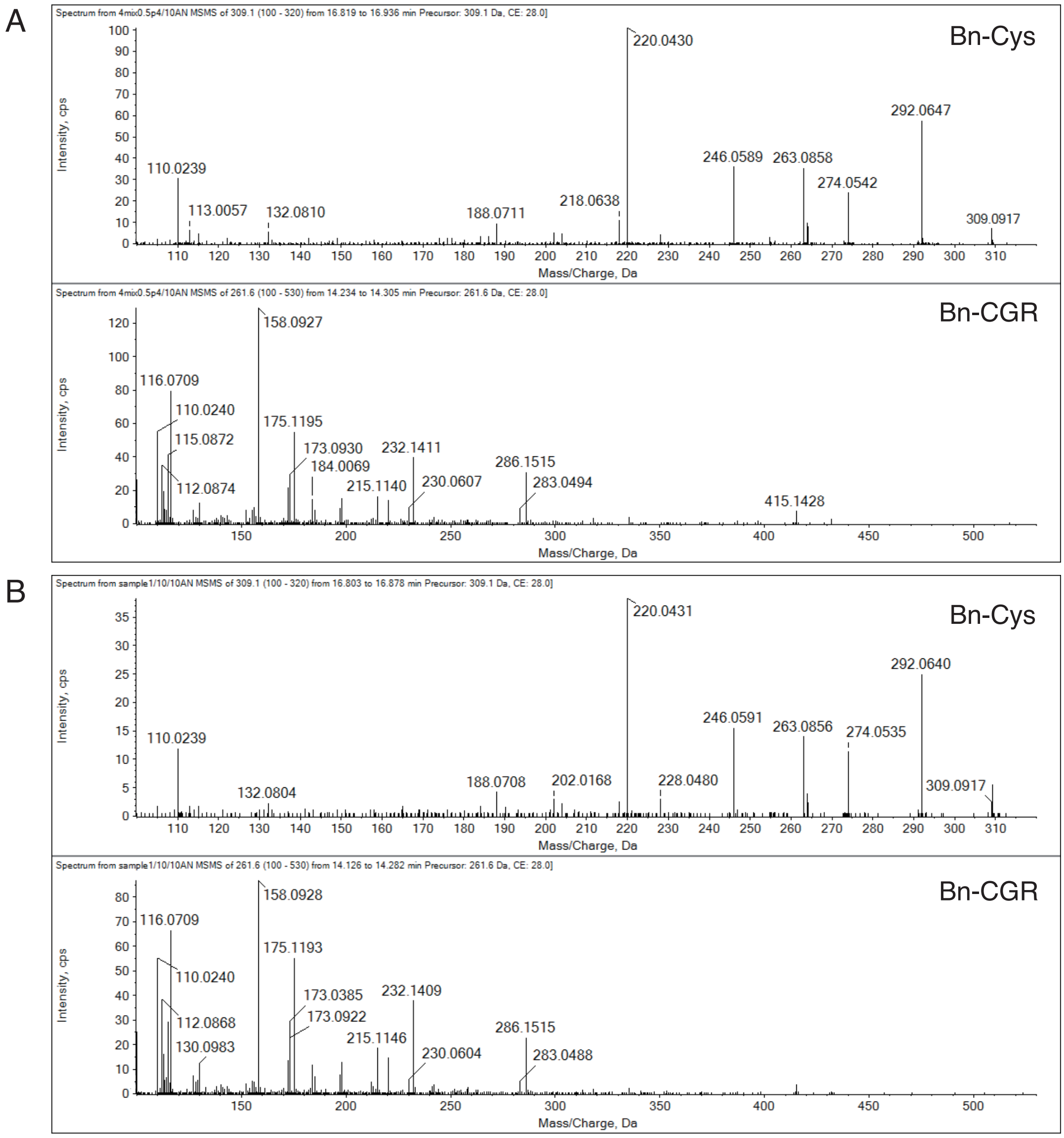

Supplementary Figure S8. Product ion spectra obtained from the MS/MS measurement for Bn-Cys or Bn-CGR. The precursor ions were set to *m/z* 309.1 for Bn-Cys and *m/z* 261.6 for Bn-CGR.

A. The standard mixed solution (0.5 μmol/L)

B. The T34Q-X sample solution dissolved in acetonitrile/water (10:90). The derivatised T34Q-X sample was dissolved in 100 μL of acetonitrile/water (50:50) and then diluted 10-fold using acetonitrile/water (5:95).

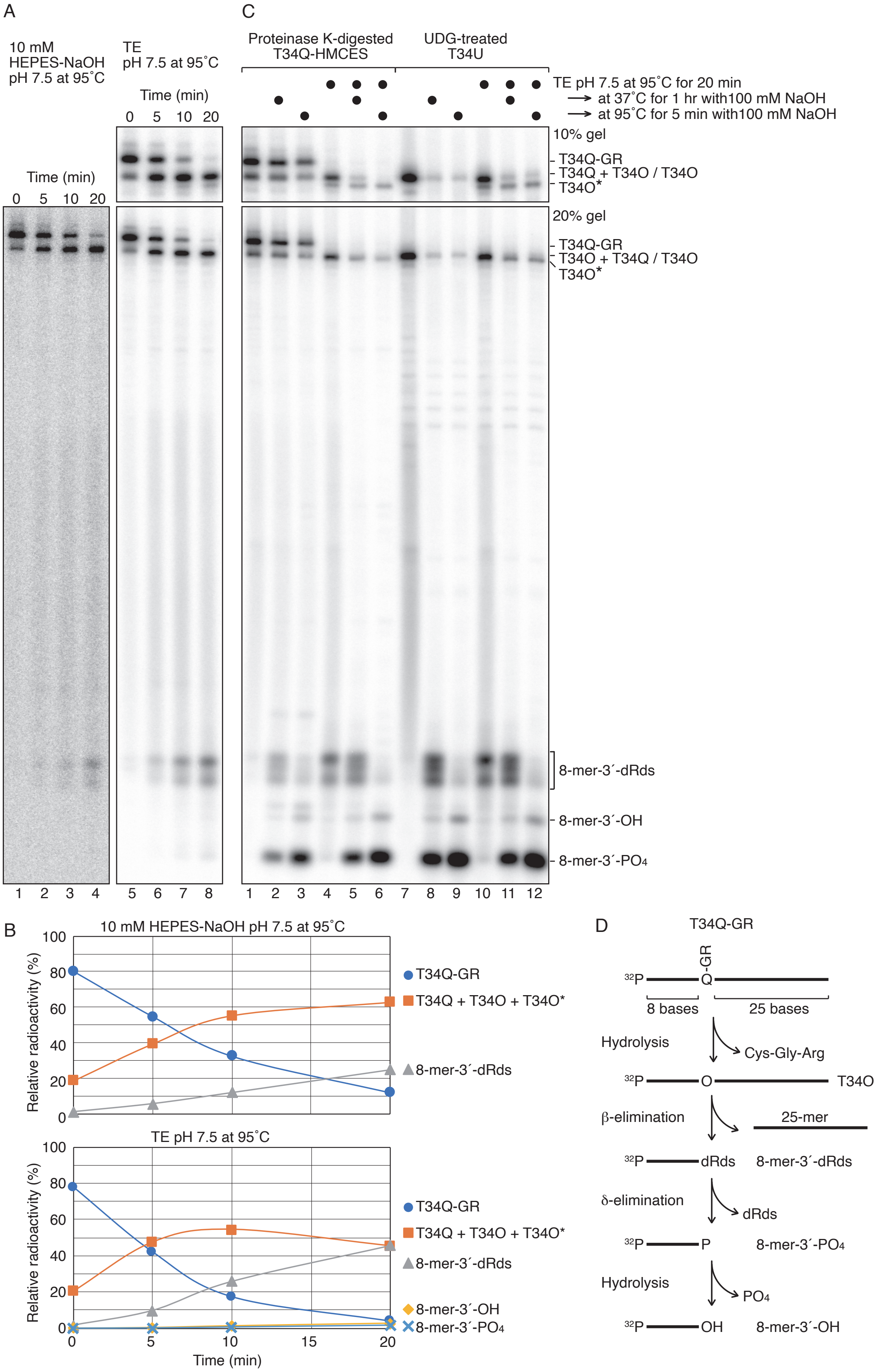
A
10 mM HEPES-NaOH pH 7.5 at 95˚C
TE pH 7.5 at 95˚C
Time (min)
0 5 10 20
C
Proteinase K-digested T34Q-HMCES
UDG-treated T34U
TE pH 7.5 at 95˚C for 20 min
→ at 37˚C for 1 hr with100 mM NaOH
→ at 95˚C for 5 min with100 mM NaOH
10% gel
T34Q-GR
T34Q + T34O / T34O
T34O*
20% gel
T34O + T34Q / T34O
8-mer-3´-dRds
8-mer-3´-OH
8-mer-3´-PO4
1 2 3 4 5 6 7 8
1 2 3 4 5 6 7 8 9 10 11 12
B
10 mM HEPES-NaOH pH 7.5 at 95˚C
TE pH 7.5 at 95˚C
Relative radioactivity (%)
T34Q + T34O + T34O*
Time (min)
D
Q-GR
8 bases
25 bases
Hydrolysis
Cys-Gly-Arg
β-elimination
25-mer
δ-elimination
dRds
PO4
OH

Supplementary Figure S9. Chemical stability of 4-carboxythiazolidine adducts.

A. Heat-instability of the Q-GR adduct. The proteinase K-digested T34Q-HMCES, a mixture of T34Q-GR (76%), T34Q (17%), and T34O (7%), was 5´-^{32}P-labelled and incubated in 10 mM HEPES-NaOH pH 7.5 (lanes 1-4) or TE (10 mM Tris-HCl pH 7.5, 1 mM EDTA) (lanes 5-8) at 95°C for the indicated time and resolved by 10% (upper panel) and 20% (lower panels) urea-PAGE.

B. Relative radioactivity of each fraction measured in 20% urea-PAGE gels in A. In 10 mM HEPES-NaOH, 8-mer-3´-OH and 8-mer-3´-PO_4 were not detected. dRds indicates deoxyribose derivatives generated by β-elimination.

C. Stability of 4-carboxythiazolidine adducts under the alkaline condition. With or without prior heat-treatment in TE at 95°C for 20 min, proteinase K-digested T34Q-HMCES and UDG-treated T34U were incubated in the presence of 100 mM NaOH at 37°C for 1 hour or 95°C for 5 min, and resolved by 10% (upper panel) or 20% (lower panel) urea-PAGE. Note that heat-treatment of T34O generated an alkaline resistant product, designated as T34O*, which migrated slightly faster than T34O.

D. Schematic of these reactions.

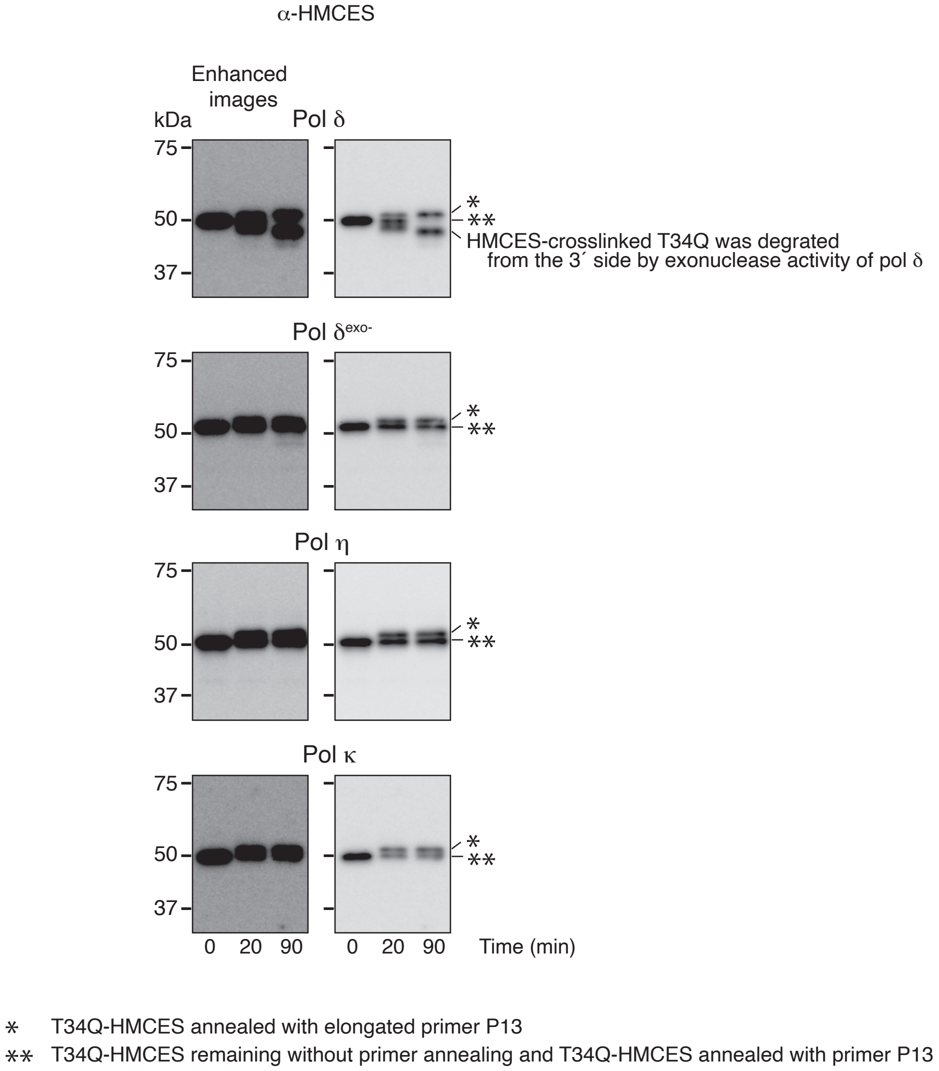

Supplementary Figure S10. Stability of HMCES-crosslinks during primer extension.

Pol δ (35 fmol), pol δ exo^{-} (75 fmol), pol η (35 fmol), or pol κ (70 fmol) was incubated with the primer-template (a mixture of T34Q-HMCES (67%) and T34O (33%)) at 30°C for the indicated times. The reaction conditions were identical to those described in Figure 3B-D middle panels. The reaction products were then incubated in the same volume of urea-SDS buffer at 37°C for 10 min, separated by 5–20% SDS-PAGE, and analysed by western blot with an anti-HMCES antibody. Because an excess amount of the template annealed with a limited amount of the primer (see also MATERIALS AND METHODS), the remaining unannealed T34Q-HMCES was not subjected to the primer extension reaction. P13-annealed T34Q-HMCES and T34Q-HMCES itself were indistinguishable by SDS-PAGE or P13 may have been denatured during sample preparation. The exonuclease activity of pol δ degraded unannealed HMCES-crosslinked T34Q

from the 3´-end. By contrast, the 3´-ends of P13-annealed T34Q-HMCES and intermediates of the primer extension were considered to be resistant to exonuclease activity because pol δ re-synthesised the degraded template using P13 as a template. The exonuclease-dependent degradation of T34Q-HMCES was confirmed by the disappearance of the degraded products following incubation with an exonuclease deficient mutant, pol δ^{exo-}. The enhanced images demonstrated that free HMCES, which should migrate slightly slower than the marker at 37 kDa (see also Figure 1H), was not detectable after 90 min incubation.

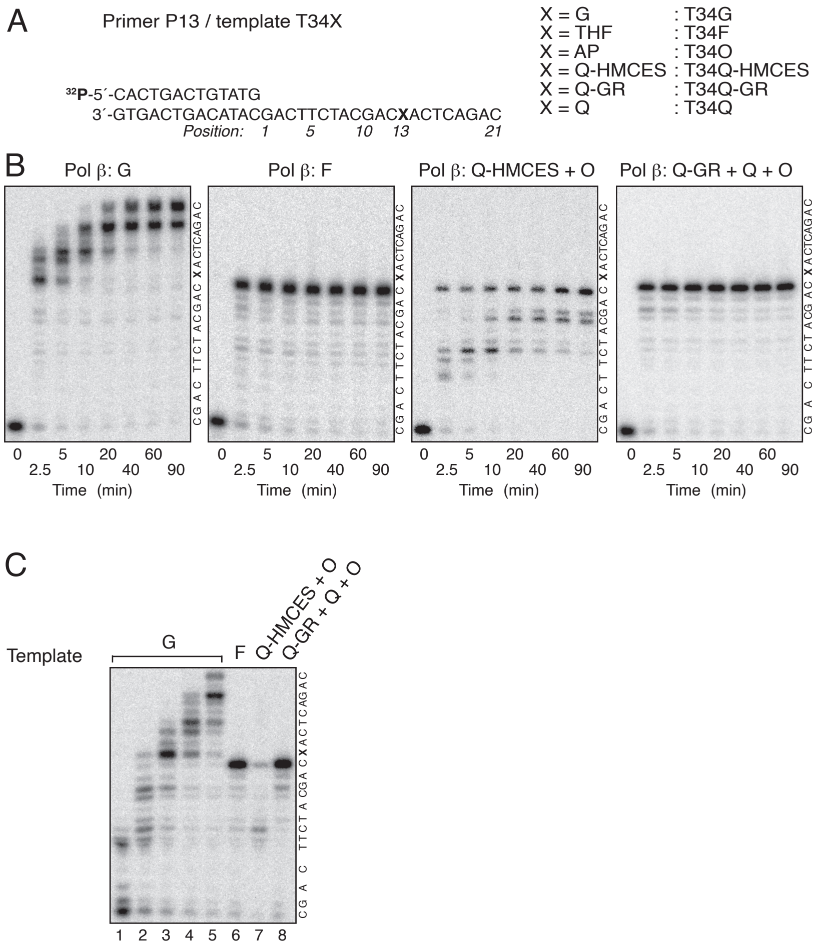

Supplementary Figure S11. Primer extension with templates containing the HMCES-crosslink or 4-carboxythiazolidine adducts by pol β.

A. Nucleotide sequences of the 5′-^{32}P-labelled primer and templates used in the assays. The assays containing the T34Q-HMCES template were performed using a template mixture of T34Q-HMCES (84%) and T34O (16%), as shown in Supplementary Figure S1D, and those containing the T34Q-GR template were performed using a template mixture of T34Q-GR (76%), T34Q (17%), and T34O (7%), as shown in Figure 1D lane 3.

B. Time courses of primer extension reactions. Pol β (82 fmol) was incubated with the indicated primer-template at 30°C for the indicated times. The reaction products were resolved by 8% urea-PAGE. The nucleotides sequences of the template strands are shown to the right of each panel.

C. The products of a 5 min incubation with the indicated templates (B) were loaded (lanes 6-8) next to the products of template T34G as size markers (lanes 1-5) to confirm the pausing positions.

A

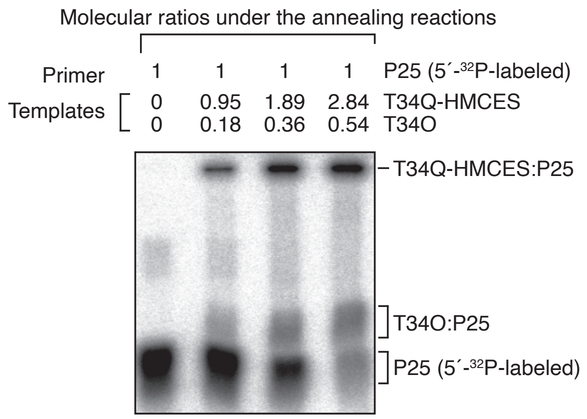

B

Annealed P25				
T34Q-HMCES:P25		8%	30%	52%
T34O:P25		7%	15%	26%
Unannealed P25	100%	85%	55%	22%

Condition used for the TLS assays

Supplementary Figure S12. Annealing reactions between P25 and a mixture of T34Q-HMCES and T34O.

A. A mixture of T34Q-HMCES and T34O was incubated with ^{32}P-labelled P25 at the indicated molecular ratios at 4°C for 10 min. The samples were then incubated in the same volume of urea-SDS buffer at 37°C for 10 min and resolved by 30% SDS-PAGE.

B. Relative radioactivity of each fraction in A.

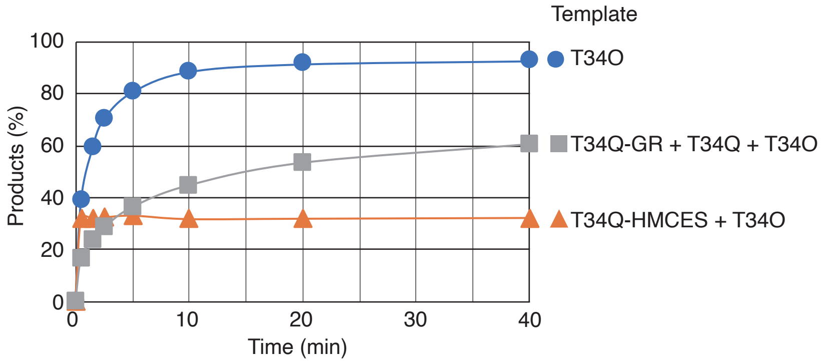

Supplementary Figure S13. 4-carboxythiazolidine adducts are not a good substrate for REV1.

The relative radioactivity of one-base elongation products was measured from the gel images in Figure 4F.

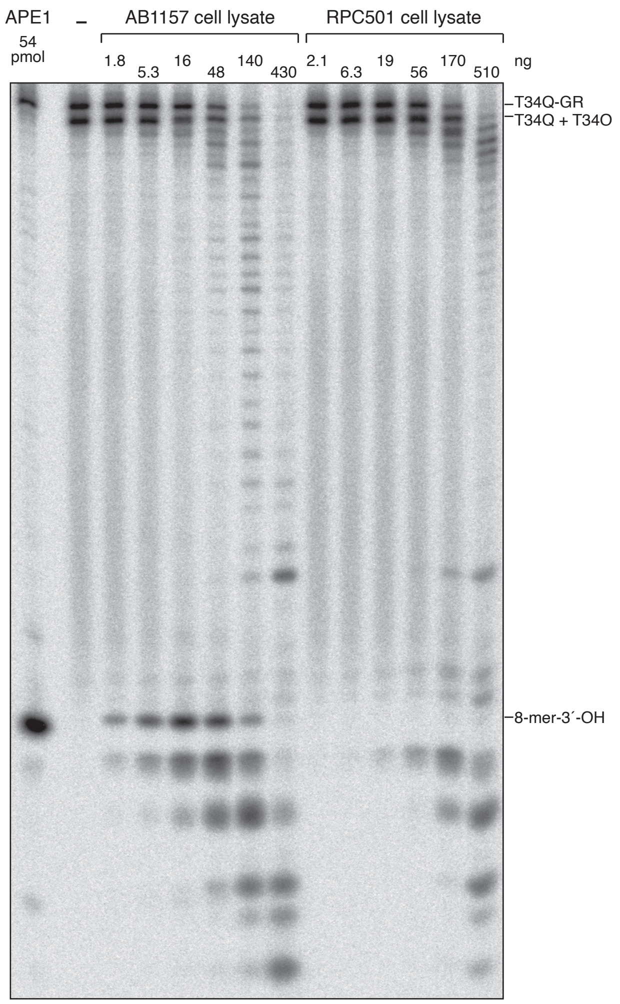

Supplementary Figure S14. Endonuclease assays of AP site and 4-carboxythiazolidine adducts under the AP endonuclease reaction condition using *E. coli* cell lysates.

The dsDNA substrates, ds31X+3 and ds34X (Figure 6A), were incubated with the indicated amounts of cell lysate and APE1, respectively, at 30°C for 10 min under the 10 mM $MgCl_2$ at pH 7.5 reaction condition. The reaction products were resolved by 20% urea-PAGE. The product of incision at 5´ to the lesions is shown as 8-mer-3´-OH.

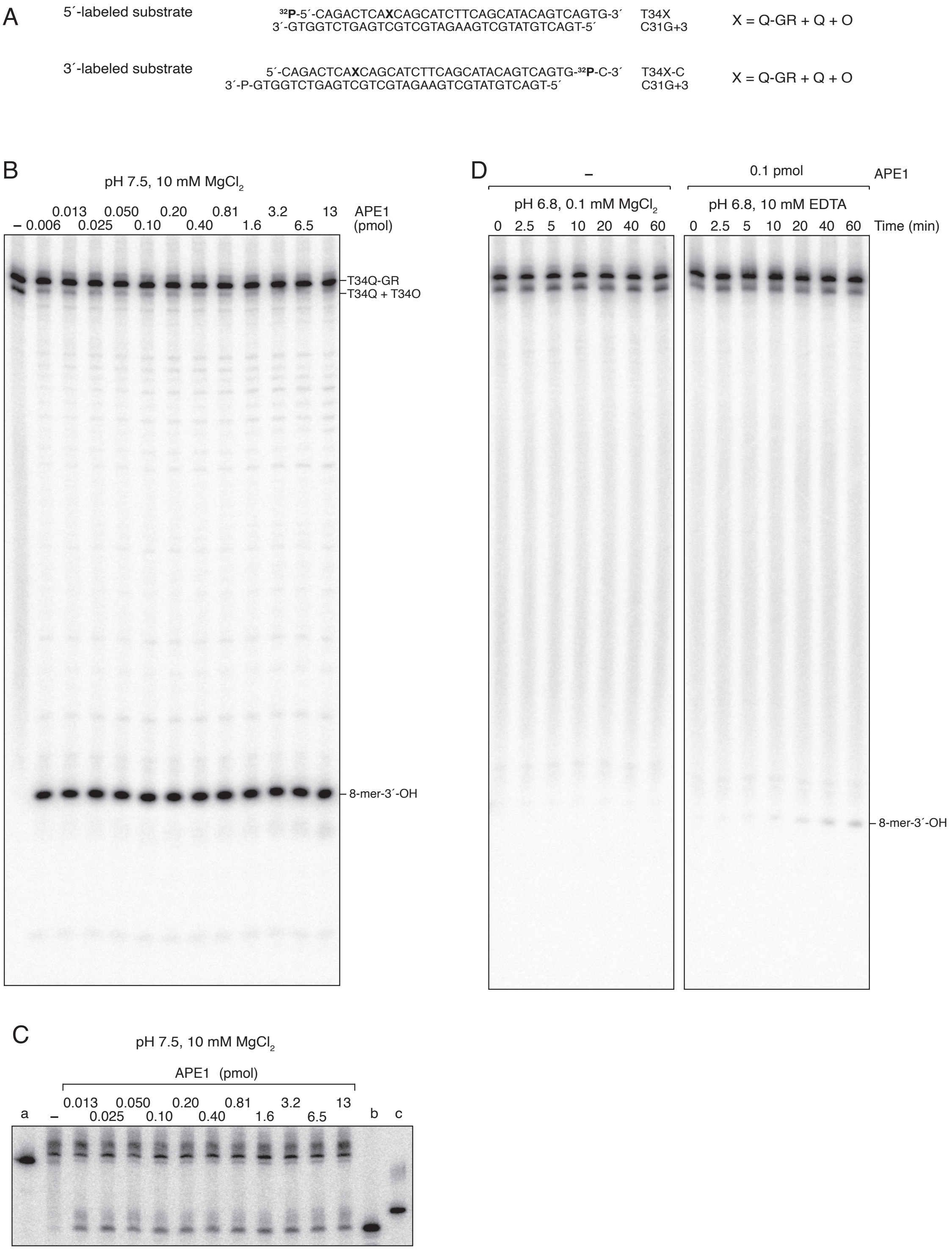

Supplementary Figure S15. Control experiments of the nuclease assays with 4-carboxythiazolidine adducts by APE1.

A. Nucleotide sequences of 5´-^{32}P-labelled substrates used in B and D and 3´-^{32}P -labelled substrates in C. An oligonucleotide mixture of T34Q-GR (76%), T34Q (17%), and T34O (7%) was labelled to generate the indicated substrates.

B. Titration of APE1. Indicated amounts of APE1 were incubated with the 5´-^{32}P-labelled substrates at 30°C for 60 min under the 10 mM $MgCl_2$ at pH 7.5 reaction condition. Reaction products were resolved by 20% urea-PAGE. The product of incision 5´ to the lesions is indicated as '8-mer-3´-OH'.

C. Titration of APE1. The indicated amounts of APE1 were incubated with the 3´-^{32}P-labelled substrates at 30°C for 60 min under the 10 mM $MgCl_2$ at pH 7.5 reaction condition. Reaction products were resolved by 20% urea-PAGE. The size markers, a, b, and c are shown in Figure 6G.

D. Stability of Q-GR adducts. The 5´-^{32}P-labelled substrates were incubated at 30°C for the indicated times under the indicated reaction conditions, in the absence of APE1 (left panel) or presence of EDTA instead of $MgCl_2$ (right panel). Reaction products were resolved by 20% urea-PAGE. The product of incision 5´ to the lesions is indicated as '8-mer-3´-OH'.

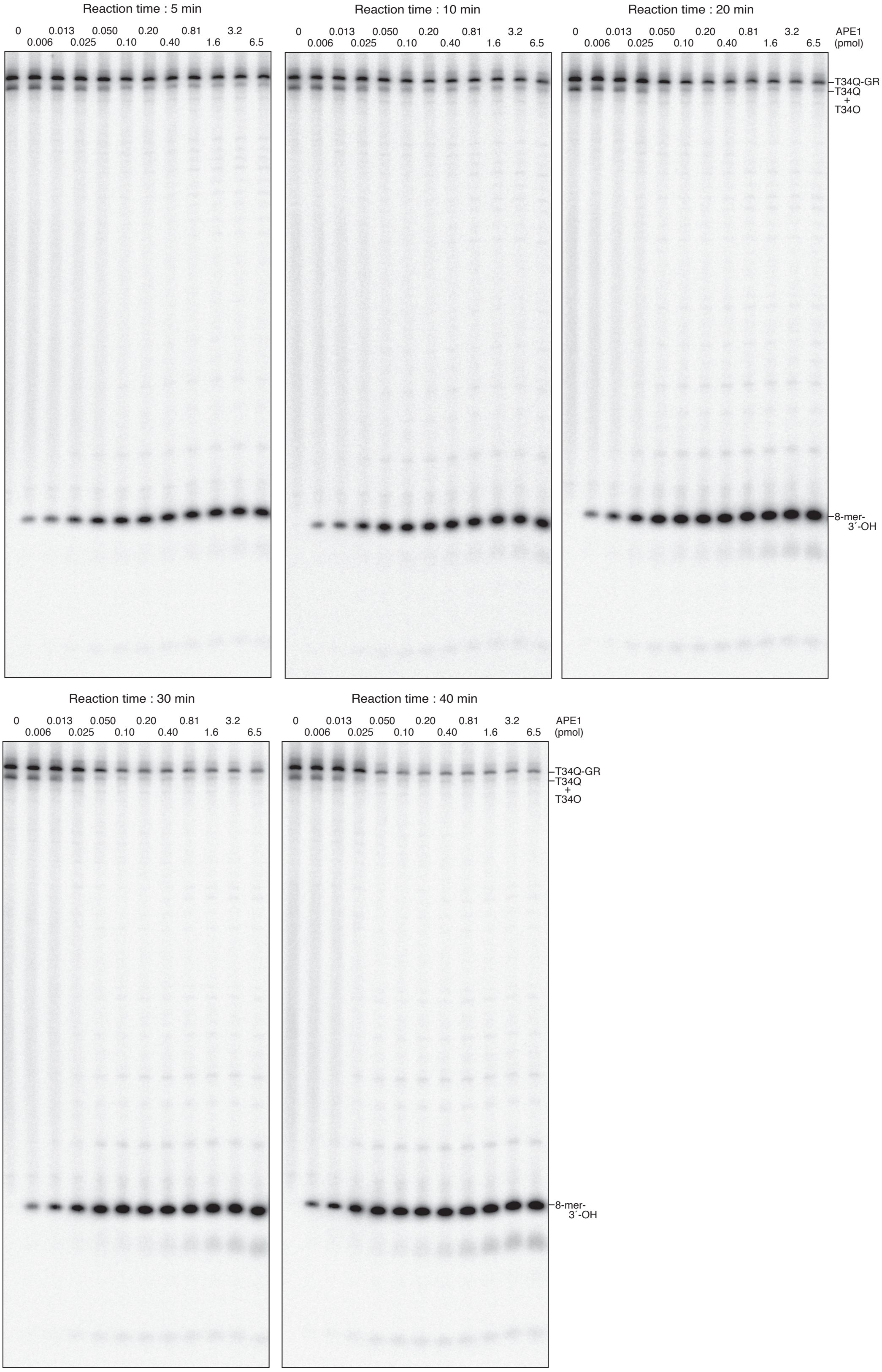

Supplementary Figure S16. The untrimmed images shown in Figure 7D.

A

5´-labeled substrate

^{32}**P**-5´-CAGACTCA**X**CAGCATCTTCAGCATACAGTCAGTG-3´ T34X
3´-GTGGTCTGAGTCGTCGTAGAAGTCGTATGTCAGT-5´ C31G+3

X = Q-GR + Q + O

3´-labeled substrate

5´-CAGACTCA**X**CAGCATCTTCAGCATACAGTCAGTG-^{32}**P**-C-3´ T34X-C
3´-P-GTGGTCTGAGTCGTCGTAGAAGTCGTATGTCAGT-5´ C31G+3

X = Q-GR + Q + O

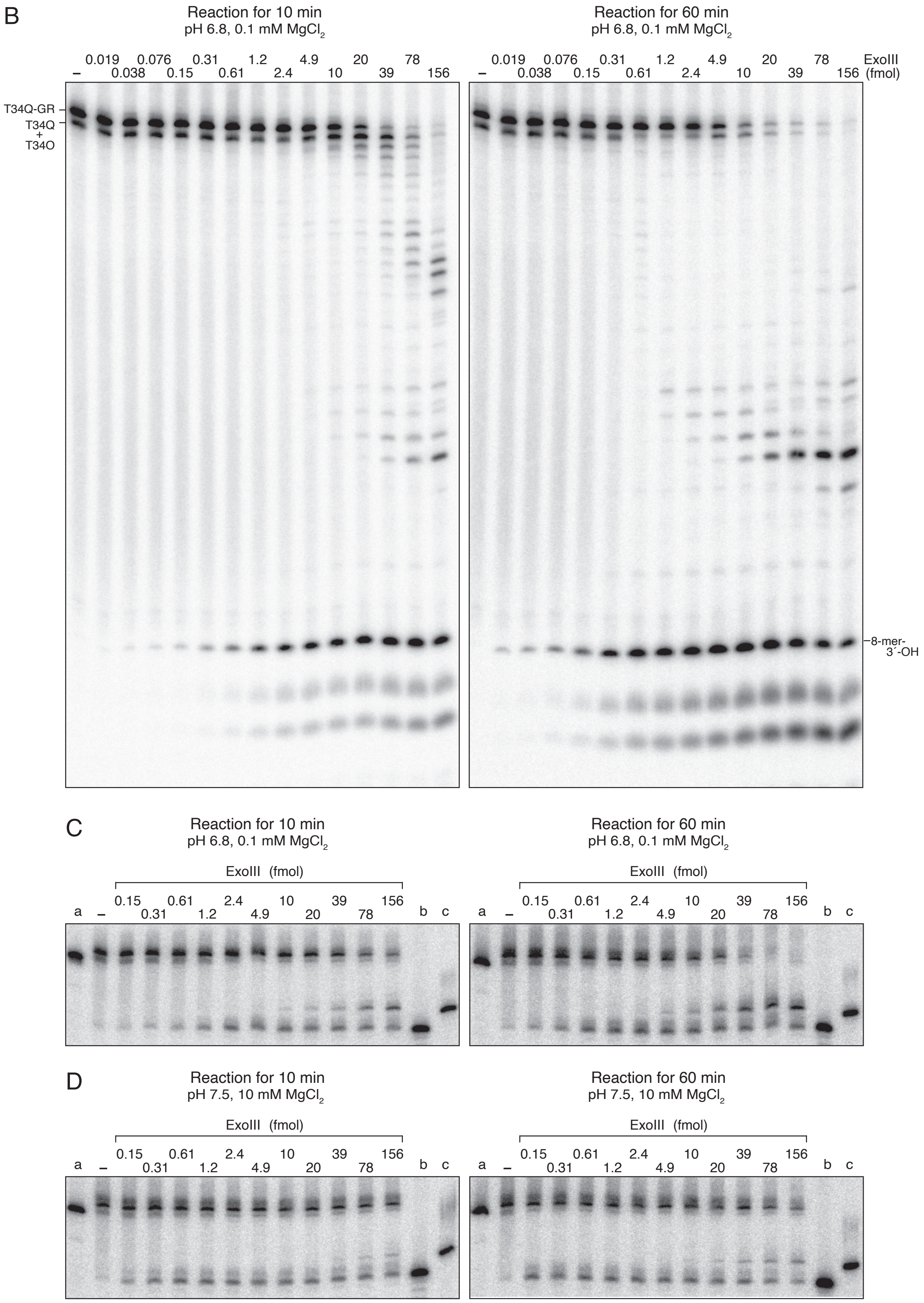

Supplementary Figure S17. Incision of 4-carboxythiazolidine adducts under the nucleotide incision reaction condition by ExoIII.

A. Nucleotide sequences of 5´-^{32}P-labelled substrates used in B and 3´-^{32}P-labelled substrates in C and D. An oligonucleotide mixture of T34Q-GR (76%), T34Q (17%), and T34O (7%) was labelled to generate the indicated substrates.

B. The 5´-^{32}P-labelled substrates were incubated with the indicated amounts of ExoIII at 30°C for 10 min (left panel) or 60 min (right panel) under 0.1 mM $MgCl_2$ at pH 6.8 reaction conditions. Reaction products were resolved by 20% urea-PAGE. The product of incision 5´ to the lesions is indicated as '8-mer-3´-OH'.

C-D. The 3´-^{32}P-labelled substrates were incubated with the indicated amounts of ExoIII at 30°C for 10 min (left panels) or 60 min (right panels) under the 0.1 mM $MgCl_2$ at pH 6.8 reaction condition (C) or under the 10 mM $MgCl_2$ at pH 7.5 reaction condition (D). Reaction products were resolved by 20% urea-PAGE. The size markers, a, b, and c are shown in Figure 6G.

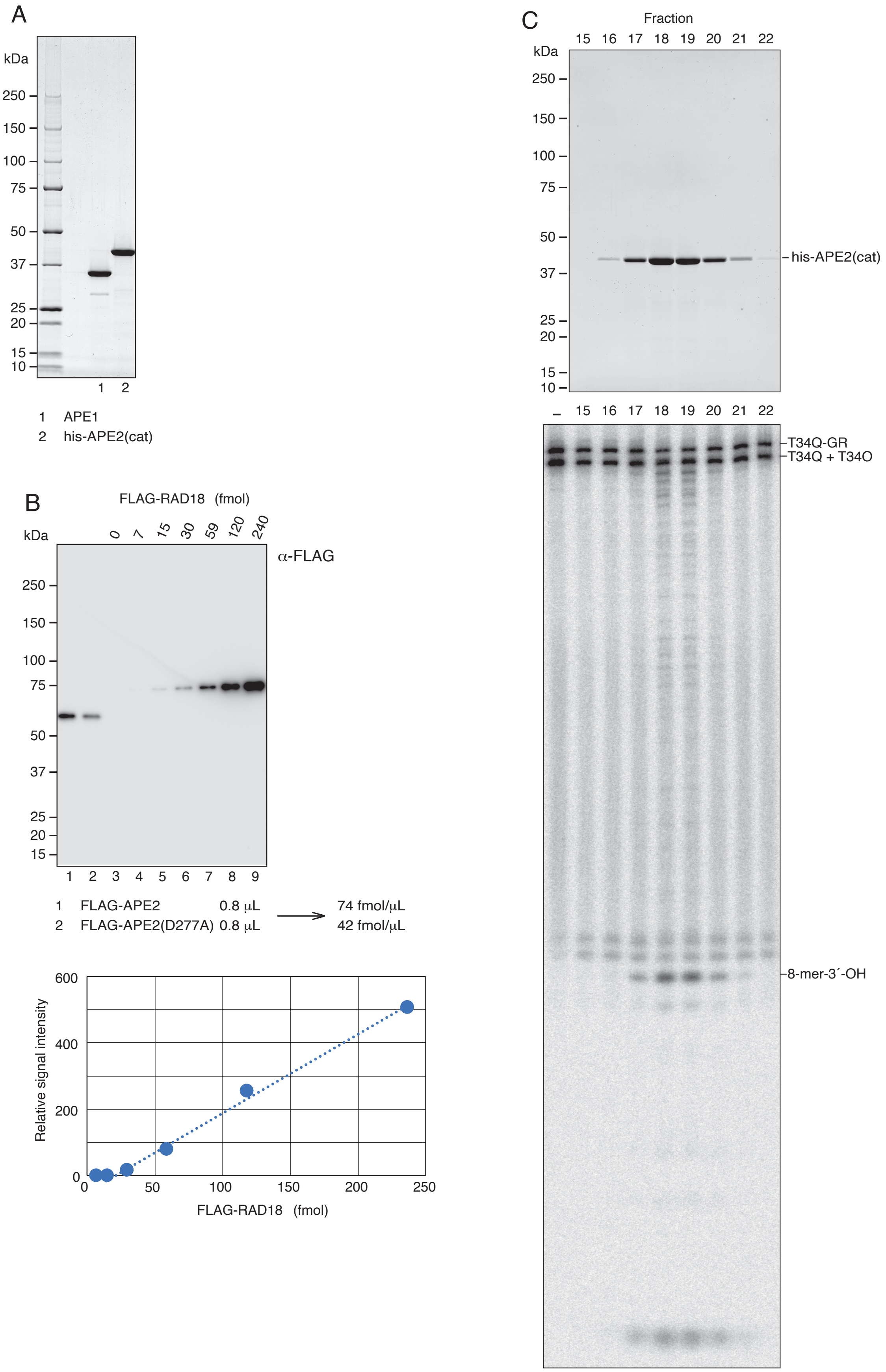

A
kDa
250
150
100
75
50
37
25
20
15
10
1 2
1 APE1
2 his-APE2(cat)
B
FLAG-RAD18 (fmol)
kDa
0 7 15 30 59 120 240
α-FLAG
250
150
100
75
50
37
25
20
15
1 2 3 4 5 6 7 8 9
1 FLAG-APE2 0.8 μL
2 FLAG-APE2(D277A) 0.8 μL
74 fmol/μL
42 fmol/μL
Relative signal intensity
600
400
200
0
0 50 100 150 200 250
FLAG-RAD18 (fmol)
C
Fraction
15 16 17 18 19 20 21 22
kDa
250
150
100
75
50
37
25
20
15
10
his-APE2(cat)
– 15 16 17 18 19 20 21 22
T34Q-GR
T34Q + T34O
8-mer-3´-OH

Supplementary Figure S18. Purified human AP endonucleases.

A. SDS-PAGE analysis of purified recombinant proteins. The indicated proteins (1 μg each) were analysed by 5–20% SDS-PAGE and stained with CBB.

B. Quantification of partially purified FLAG-APE2. Cell lysates transfected with respective expression vectors were subjected to immunoprecipitation with anti-FLAG antibody and eluted with a FLAG peptide. The indicated samples (0.8 μL each, lanes 1 and 2) were examined by western blot with an anti-FLAG antibody. The indicated amounts of purified recombinant FLAG-RAD18-his-RAD18-RAD6 ternary complex (44) were loaded side by side as a standard (lanes 3-9). The concentrations of FLAG-APE2 and FLAG-APE2(D277A) proteins were estimated from the standard curve of FLAG-RAD18.

C. Elution profile of his-APE2(cat) from gel filtration chromatography.

Upper panel. Indicated fractions (1 μL) eluted from the Superdex 200 column were analysed by 5–20% SDS-PAGE and stained with CBB.

Lower panel. Indicated fractions (1.6 μL) were subjected to an exonuclease assay in the presence of 50 μM $CoCl_2$ with a substrate mixture of T34Q-GR (50%), T34Q (10%), and T34O (40%) labelled with ^{32}P and annealed with an excess amount of C23G+11, as shown in Figure 8A. The reaction products were resolved by 20% urea-PAGE. The product of incision at 5´ to the lesions is shown as the 8-mer-3´-OH.

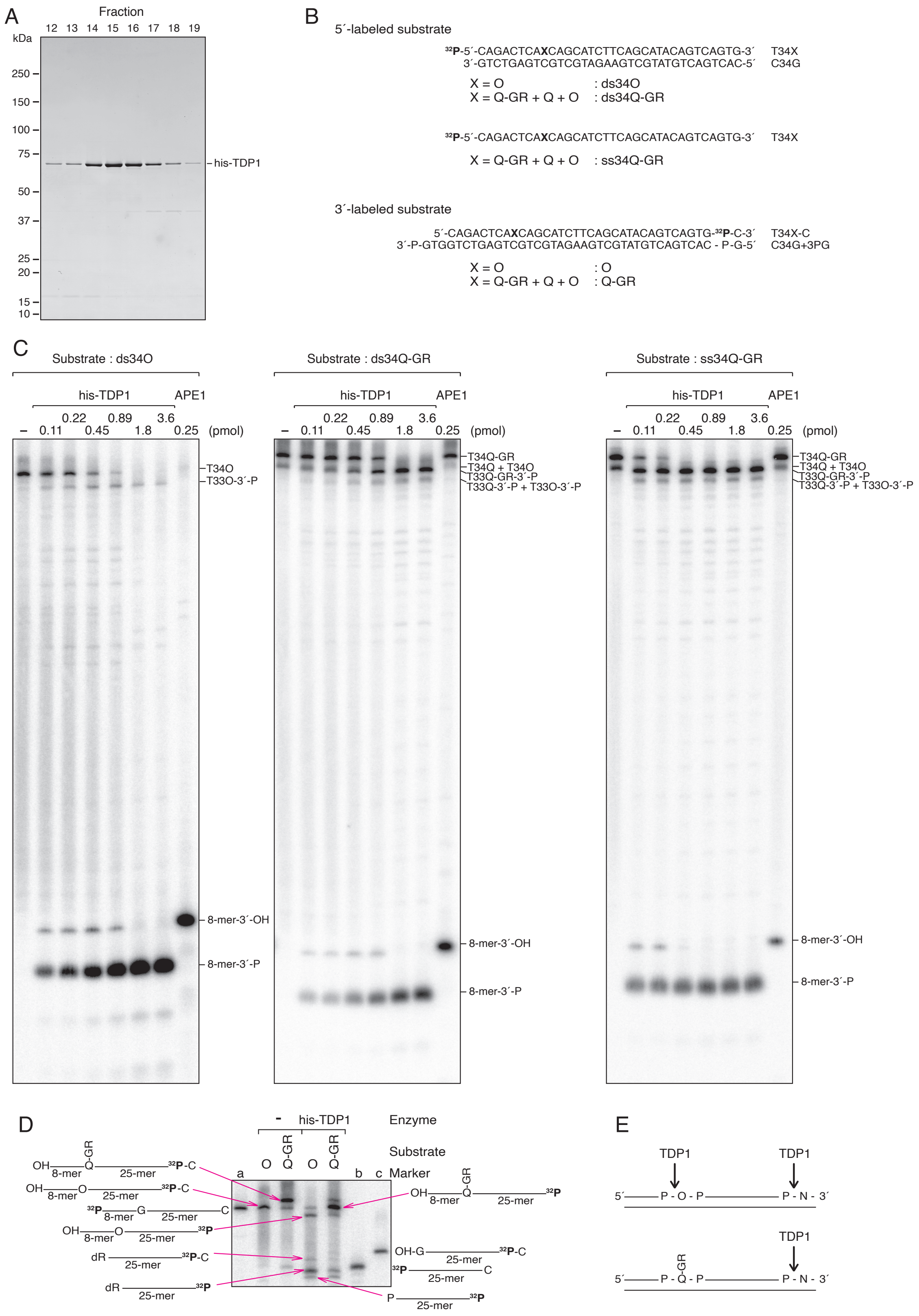

Supplementary Figure S19. Phosphodiesterase assays of TDP1

A. Elution profile of his-TDP1 from gel filtration chromatography. The indicated fractions (2 μL) eluted from gel filtration chromatography were analysed by 5–20% SDS-PAGE and stained with CBB.

B. Nucleotide sequences of 5´-^{32}P-labelled substrates used in C and 3´-^{32}P-labelled substrates in D. T34O and an oligonucleotide mixture of T34Q-GR (76%), T34Q (17%), and T34O (7%) were labelled to generate the indicated substrates.

C. The 5´-^{32}P-labelled substrates were incubated with the indicated amounts of his-TDP1 at 30°C for 30 min under the phosphodiesterase assay condition. To generate the marker of the incision product, 8-mer-3´-OH, APE1 was reacted with indicated substrates under the 10 mM $MgCl_2$ at pH 7.5 reaction condition. Note that APE1 has weak activity for the ssDNA substrate, ss34Q-GR (right panel). Reaction products were resolved by 20% urea-PAGE. A product of incision at the AP site by TDP1 is indicated as '8-mer-3´-P'. Excision products of the substrates, T34Q-GR, T34Q and T34O, generated by removal of 3´-mononucleoside moiety are referred to as 'T33Q-GR-3´-P', 'T33Q-3´-P' and 'T33O-3´-P', respectively.

D. The 3´-^{32}P-labelled substrates were incubated with his-TDP1 (3.6 pmol) at 30°C for 30 min under the phosphodiesterase assay condition. Reaction products were resolved by 20% urea-PAGE. The size markers, a, b, and c are shown in Figure 6G.

E. Schematic of the incision sites by TDP1.
